# A Biobased Epoxy Vitrimer with Dual Relaxation Mechanism: A Promising Material for Renewable, Reusable, and Recyclable Adhesives and Composites

**DOI:** 10.1021/acssuschemeng.4c00205

**Published:** 2024-04-04

**Authors:** Pere Verdugo, David Santiago, Silvia De la Flor, Àngels Serra

**Affiliations:** † Technology Center of Catalonia - Chemical Technologies Unit, Eurecat, c/Marcel·lí Domingo 2, 43007 Tarragona, Spain; ‡ Department of Analytical and Organic Chemistry, Universitat Rovira i Virgili, c/Marcel·lí Domingo 1, 43007 Tarragona, Spain; § Department of Mechanical Engineering, Universitat Rovira i Virgili, Av. Països Catalans 26, 43007 Tarragona, Spain

**Keywords:** vanillin, epoxy, vitrimers, disulfide, imine, composite, adhesion

## Abstract

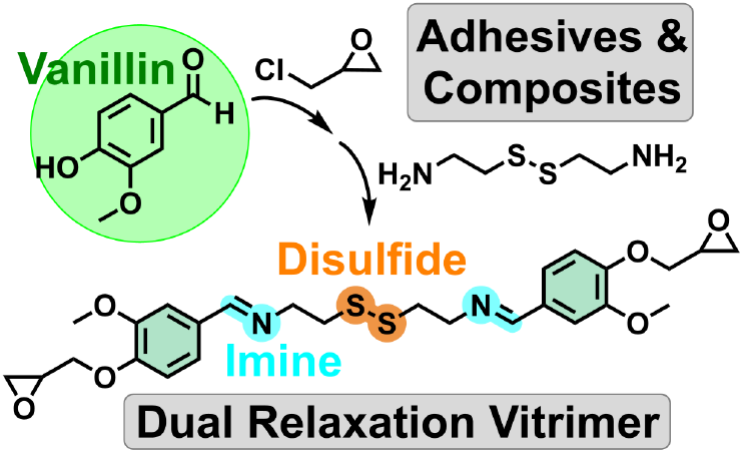

This study presents the synthesis of a novel biobased epoxy monomer derived from vanillin and cystamine, incorporating imine and disulfide exchangeable groups within its structure. A series of epoxy-based vitrimers with two simultaneous exchange relaxation processes have been produced using this monomer. These exchange mechanisms operate without the need for any catalyst. Four different amine curing agents have been employed to achieve vitrimers with glass transition temperatures around 100 °C and excellent thermal stability. Through dynamic-mechanical analyses, thermomechanical properties and vitrimeric characteristics have been investigated, revealing remarkably fast stress relaxation at relatively low temperatures without significant creep below the glass transition temperature. Leveraging the dual exchange mechanism, the chemical degradability of these vitrimers has been explored through two accessible methodologies, and the material’s reformation after degradation has been demonstrated in both cases. Furthermore, the material has been mechanically recycled, maintaining almost the same properties. Finally, these materials have been used to fabricate and recycle carbon-fiber-reinforced composite material and reversible adhesives, showcasing their promising potential applications.

## Abstract 2

The obtained material is synthesized only using renewable resources and solvents, with an easy synthesis, and the capability of being reused and recycled.

## 1 Introduction

Thermosetting materials are of great interest due to their outstanding mechanical and thermal properties.^1^ These properties result from the three-dimensional covalent structure that presents this type of material. For this reason, thermosetting materials are nowadays used in a broad range of applications, such as adhesives, coatings, composites, the aerospace industry, and electronic materials.^2^ The main drawback of thermosetting materials is that their permanent cross-linked structure does not allow reforming or recycling once the material is cured. To overcome this inherent disadvantage, dynamic covalent bonds can be introduced into the structure to obtain the commonly named covalent adaptable networks (CANs).^3^

Kloxin and Bowman^4^ defined CANs as polymer networks in which the cross-linking points of the thermosetting material can undergo reversible rearrangement reactions and thus provide a macroscopic flow and stress relaxation. Different reversible exchange reactions have been reported so far for the preparation of vitrimers, such as transesterification, transamination, disulfide exchange, siloxane equilibrium, amine–urea exchange, and transcarbamoylation.^5^ Some of them require the presence of a catalyst, such as hydroxy ester or thiourethane, and others do not, such as imine and disulfide groups.^6^

Nowadays, the imine and disulfide groups are attracting great interest, not only because of their fast stress relaxation times but also because of their ease of recycling, both mechanically and chemically.^7,8^ A common route to obtain imine-containing epoxy vitrimers is through vanillin. In this sense, several vanillin-based epoxy monomers can be found in the literature, mainly because it is a cheap and abundant natural molecule with an aldehyde and hydroxyl group, which can be used for many purposes.^9^ Memon et al.^7^ prepared an imine hardener for epoxy resins curing from vanillin and isophorone diamine. Moreover, thanks to the aldehyde moiety, several authors used diamines to link two molecules of vanillin and obtain a molecule with diimine groups. Roig et al.^10^ prepared an epoxy monomer from vanillin and 4,4′-oxidianiline, which was cross-linked with amines, whereas Zhao et al.^11^ synthesized a diphenol–diimine from vanillin and 4,4-diaminodiphenylmethane, which was used to cure epoxidized soybean oil.

Commonly, to introduce disulfide bonds within the epoxy-based vitrimer network, 2,2′-diaminodiphenyl disulfide^12^ or 4,4′-diaminodiphenyl disulfide^13^ is used as a curing agent. Some studies can be found in the literature in which the authors reported vitrimers with dual relaxation mechanisms. For instance, Chen et al.,^14^ Sun et al.,^15^ Wang et al.,^16^ Konuray et al.,^17^ and Vilanova-Pérez et al.^18^ studied vitrimers with disulfide metathesis and transesterification. Introducing imine and disulfide exchangeable groups has also been studied in detail thanks to the aldehyde group of vanillin and the disulfide bond of 4-aminophenyl disulfide.^19−22^ Other examples of vitrimers with dual relaxation mechanisms are the studies published by Fortmant et al.,^23^ in which they prepared a polyhydroxyurethane with transcarbamoylation and disulfide metathesis, and by Roig et al.^24^ The latter contained three different dynamic bonds, esters, disulfide, and β-aminoesters, and the material is partially based on biobased resources, such as eugenol and cystamine.

Another aspect to consider is the renewability of the raw materials used for its synthesis. Some of the aforementioned studies are based on DGEBA and diaminodiphenyl disulfide, which are fossil-based. To fulfill the principles of green chemistry, it is mandatory to avoid the use of fossil-based feedstock and to use those that can be obtained in a sustainable manner.^25^ Nowadays, a great variety of compounds can be obtained from renewable resources such as vegetable oils^26^ and carbohydrates.^27^ However, from the socio-economical point of view, it is important that the use of these natural resources will not enter in conflict with other human activities, such as the food industry, that could diminish the availability of these raw materials and, therefore, causing an increase in their price.^28^ The use of biomass-derived waste is a feasible option for the obtention of these chemicals. Lignin can be derived from agricultural and forestry wastes, and from it can be prepared different valuable compounds, such as vanillin or syringaldehyde.^29^

Here, we propose an epoxy monomer with imine and disulfide groups, derived from renewable sources, that can be used to prepare epoxy-based vitrimers. The differential feature concerning the present state-of-the-art is that the incorporation of aliphatic imine and disulfide groups, involved in the exchange reactions, is part of the epoxy monomer, so that one can use any curing agent suitable for epoxy resins to obtain a vitrimer. The use of aliphatic amines to form the imine moieties produce a structure easier to hydrolyze under mild acidic conditions.

In this work, vitrimers were obtained by curing with four different amines, showing extremely fast relaxation times and easy degradability by two different methods and showing as well very good thermomechanical properties. Furthermore, the monomer was synthesized from renewable vanillin, which can be obtained from lignin,^9^ the biobased cystamine and epichlorohydrin, which, nowadays, can also be obtained from renewable sources.^30,31^ Moreover, the monomer was synthesized using 2-methyltetrahydrofuran as renewable solvent and was cured with the renewable isophorone diamine, among other curing agents. Finally, the excellent performance of these materials was demonstrated through their application as reversible adhesives and reconformable and recyclable composites.

## 2 Experimental Part

### 2.1 Materials

The following chemicals were purchased from Sigma-Aldrich (St. Louis, MO, USA): cystamine dihydrochloride (96%), which was previously neutralized with a 3 M sodium hydroxide solution and extracted with ethyl acetate to obtain the free base, 1,2-diaminocyclohexane (DAC, 99%, mixture of *cis* and *trans*), (±)-epichlorohydrin (ECH, ≥99%), and 4-hydroxy-3-methoxy benzaldehyde (vanillin, 99%). 5-Amino-1,3,3-trimethylcyclohexanemethylamine (isophorone diamine, (IPDA, 99%, mixture of *cis* and *trans*)) and benzyl triethylammonium chloride (BTEAC, 98%) were purchased from Acros Organics (Geel, Belgium). Sodium hydroxide (pellets, 97%), 2-methyltetrahydrofuran (Me-THF, 99+%) anhydrous, *m*-xylylene diamine (*m*-XDA, 99%), and tris(2-aminoethyl) amine (TREN, 97%) were purchased from Thermo Fisher Scientific (Waltham, MA, USA). Anhydrous magnesium sulfate (99.5%, powder) was purchased from Alfa Aesar (Haverhill, MA, USA). Absolute ethanol (EtOH, reagent grade >99.8%) and ethyl acetate (reagent grade, 99%) were purchased from VWR Chemicals (Radnor, PA, USA). All the reagents were used as received unless otherwise specified.

### 2.2 Synthesis of Vanillin Glycidyl Ether (VGE)

The vanillin glycidyl ether (VGE) was synthesized following a modification of a reported procedure.^32^ 100.0 g (0.66 mol) of vanillin, 258 mL (3.30 mol) of epichlorohydrin, and 7.5 g (0.03 mol) of benzyltriethylammonium chloride (BTEAC) were introduced into a two-necked round-bottom flask equipped with a magnetic stirrer and a condenser. The mixture was stirred at 80 °C for 3 h. Next, the mixture was cooled down in an ice–water bath, and 29.0 g (0.73 mol) of sodium hydroxide, solubilized in 115 mL of water, was added dropwise into the reaction mixture. Once added, the temperature was let to rise to room temperature and left under stirring for an additional 2 h. After the completion of the reaction, a white solid precipitated from the reaction mixture, the solid was filtered through a sintered glass funnel, and the solid was washed with distilled water (5 × 300 mL) and finally washed with cold ethanol (3 × 200 mL). The solid was transferred into a 500 mL round-bottomed flask and was dried under vacuum. The pure product was obtained with 78% yield (106.8 g). mp (DSC) = 101.0 °C.

ESI-MS, exact mass *m*/*z* [M+H^+^] = 209.0809 (theoretical mass: 209.0809).

^1^H NMR (CDCl_3_, 400 MHz, TMS, δ ppm): 9.88 (s, 1H, −CHO), 7.42 (m, 2H, Ar), 7.03 (d, ^4^*J* = 8 Hz, 1H, Ar), 4.38 (dd, ^2^*J* = 12 Hz, ^3^*J* = 4 Hz, 1H, CH_2_O–Ar), 4.10 (m, 1H, CH_2_O–Ar), 3.93 (s, 3H, OCH_3_), 3.42 (m, 1H, CH–CH_2_O), 2.93 (m, 1H, CH_2_O), 2.78 (m, 1H, CH_2_O).

^13^C NMR (CDCl_3_, 100.6 MHz, δ ppm): 190.8 (−CHO), 153.3 (Ar), 149.9 (Ar.), 130.6 (Ar), 126.4 (Ar), 112.2 (Ar•), 109.4 (Ar), 69.9 (CH_2_O–Ar), 55.9 (OCH_3_), 49.8 (CH–CH_2_O), 44.6 (CH_2_O).

### 2.3 Synthesis of Cystamine Bis(vanillin glycidyl ether) (Cyst-BVGE)

The imine derivative of vanillin glycidyl ether with cystamine (Cyst-BVGE) was synthesized as follows: 0.5039 g (3.31 mmol) of cystamine (free base) was introduced into a 50 mL round-bottomed flask equipped with a magnetic stirrer. Then, 1.3792 g (6.62 mmol) of VGE, solubilized in 40 mL of anhydrous Me-THF, was added. The mixture was kept under stirring at room temperature for 2 h. After that, the solvent was evaporated under reduced pressure, and the viscous mixture was kept at 40 °C under high vacuum overnight. The desired product was obtained as a yellowish viscous oil, which solidifies at room temperature, with quantitative conversion, and was used in the curing process without further purification.

ESI-MS, exact mass *m*/*z* [M+H^+^] = 533.1776 (theoretical mass: 533.1775).

^1^H NMR (CDCl_3_, 400 MHz, TMS, δ ppm): 8.20 (s, 2H, N=CH−), 7.40 (d, ^4^*J* = 4 Hz, 2H, Ar), 7.12 (dd, ^4^*J* = 4 Hz, ^3^*J* = 8 Hz, 2H, Ar), 6.91 (d, ^3^*J* = 8 Hz, 2H, Ar), 4.29 (dd, ^2^*J* = 12 Hz, ^3^*J* = 4 Hz, 2H, CH_2_O–Ar), 4.05 (m, 1H, CH_2_O–Ar), 3.92 (s, 6H, OCH_3_), 3.87 (m, 4H, =N–CH_2_−), 3.40 (m, 2H, CH–CH_2_O), 3.05 (t, ^3^*J* = 8 Hz, CH_2_–S), 2.92 (m, 2H, CH_2_O), 2.75 (m, 2H, CH_2_O).

^13^C NMR (CDCl_3_, 100.6 MHz, δ ppm): 162.1 (N=CH−), 150.4 (Ar), 149.7 (Ar), 129.9 (Ar), 123.1 (Ar), 112.7 (Ar), 109.3 (Ar), 70.0 (CH_2_O–Ar), 60.1 (=N–CH_2_−), 56.0 (OCH_3_), 50.1 (CH–CH_2_O), 44.9 (CH_2_O), 39.6 (CH_2_–S).

### 2.4 Preparation of the Formulations

**Scheme 1:**
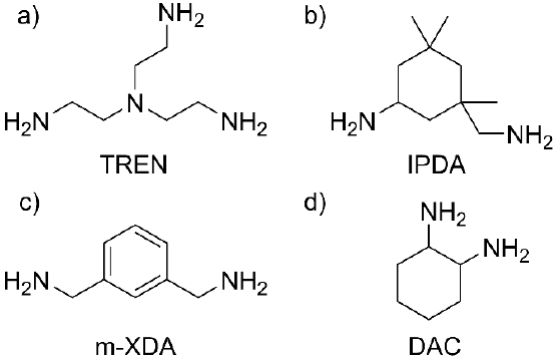
Chemical Structure of (a) Tris(2-aminoethyl) Amine (TREN), (b) Isophorone Diamine (IPDA), (c) *m*-Xylylene Diamine (*m*-XDA), and (d) 1,2-Diamino Cyclohexane (DAC)

The synthesized Cyst-BVGE was formulated with isophorone diamine (IPDA), *m*-xylylene diamine (*m*-XDA), and 1,2-diaminocyclohexane (DAC) as diamines and tris(2-aminoethyl) amine (TREN) as triamine (Scheme 1). All the formulations were prepared in stoichiometric proportions of epoxide:amine groups (2:1 mol:mol). The composition of the formulations prepared is detailed in Table 1. As a typical preparation of a sample, 2.1400 g (4.02 mmol) of Cyst-BVGE was transferred into a 50 mL round-bottomed flask equipped with a Teflon-coated magnetic stirrer. Then, 0.1958 g (1.34 mmol) of TREN was weighed directly into the flask. Due to the high viscosity of the monomer, the monomer/amine mixture was prepared into a round-bottomed flask equipped with a magnetic stirrer, introduced into a preheated thermostatic bath at 45 °C, and stirred under a high vacuum for the elimination of the trapped air to avoid the appearance of bubbles in the final material. After 5 min under vacuum, the mixture became completely homogeneous, and the formulation was poured into a preheated Teflon mold with the help of a spatula, and the formulation was cured in an oven for 2 h at 100 °C, 2 h at 140 °C, and 1 h at 160 °C. Samples were polished with sandpaper until obtaining the desired dimensions.

**Table 1:** Monomer/Amine Weight Proportions in the Prepared Formulations

formulation	Cyst-BVGE (g)	amine (g)
Cyst-BVGE/TREN	2.1400	0.1958
Cyst-BVGE/IPDA	1.1300	0.1810
Cyst-BVGE/*m*-XDA	1.0188	0.1306
Cyst-BVGE/DAC	1.1500	0.1238

### 2.5 Monomer Characterization

All the synthesized products and the products obtained after the chemical degradation were characterized by NMR spectroscopy (^1^H NMR and ^13^C NMR) using a Varian VNMR-S400 NMR spectrometer (Agilent Technologies, Santa Clara, CA, USA). CDCl_3_ was used as the solvent. All chemical shifts were quoted on the δ scale in part per million (ppm) using the residual solvent peak as the reference (^1^H NMR: CDCl_3_ = 7.26 ppm and ^13^C NMR: CDCl_3_ = 77.16 ppm). The exact mass of the synthesized products was analyzed on a Thermo Scientific Orbitrap IDX Tribrid mass spectrometer with a HESI interface, in line with a Vanquish UHPLC. No column was used. ACN/H_2_O (A) and MeOH/formic acid 0.1% (B) at 50% each were used for elution. The flow was set to 0.15 mL/min. An injection volume of 1 mL was used.

### 2.6 Thermal Characterization

The study of the curing process was performed by differential scanning calorimetry (DSC) using a Mettler-Toledo DSC3+ (Columbus, OH, USA) instrument calibrated using indium (heat flow calibration) and zinc (temperature calibration) standards. Samples of approximately 8–10 mg were placed in aluminum pans with pierced lids and analyzed under a flow of N_2_ at 50 mL·min^–1^. The curing process was studied in nonisothermal mode at 10 °C·min^–1^ from 30 to 250 °C. The glass transition temperature (*T*_g_) of the cured samples was determined in dynamic scans at 50 °C·min^–1^ from −20 to 180 °C.

The thermal stability of cured samples was studied by thermogravimetric analysis (TGA), using a Mettler-Toledo TGA 2 thermobalance (Columbus, OH, USA). All the experiments were performed under a flow of N_2_ at 50 mL·min^–1^. Pieces of cured samples of a mass of approximately 10 mg were degraded between 30 and 600 °C at a heating rate of 10 °C·min^–1^. The thermal stability was also studied in isothermal mode at 160 °C for 3 h.

### 2.7 Thermomechanical Characterization

Thermomechanical properties were measured using a TA Instruments Discovery DMA 850 (New Castle, DE, USA) equipped with a tension film clamp. Prismatic rectangular samples of about 30 mm × 6 mm × 1.5 mm were analyzed at 1 Hz, 0.1% strain, and from 30 to 150 °C at 3 °C·min^–1^. The storage modulus at glassy state () and at rubbery state () were obtained at *T*_g_ – 50 °C and at *T*_g_ + 50 °C, respectively. The *T*_g_s were determined from the maximum of the peak of tan δ.

### 2.8 Stress Relaxation Tests

Stress relaxation tests were carried out using a TA Instruments Discovery DMA 850 (New Castle, DE, USA) equipped with a film tension clamp on samples with the same dimensions as previously defined. Samples were first equilibrated at temperatures around the *T*_g_ of each formulation, then a constant strain of 1% (within the linear range) was applied to the sample, and the consequent stress level was monitored as a function of time. The process was repeated every 10 °C, up to 160 °C. The stress σ was normalized by the initial stress σ_0_, and the characteristic relaxation time τ was determined as the time necessary to relax 1/*e* of the initial stress value σ_0_. The activation energy *E*_a_ was calculated for each material by using an Arrhenius-type eq 1:
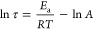
1where τ is the time needed to attain a given stress relaxation value of 1/*e*·σ_0_, *R* is the gas constant, *T* is the absolute temperature, and *A* is the pre-exponential factor.

### 2.9 Creep Experiments

Creep and recovery properties were studied using a TA Instruments Discovery DMA 850 (New Castle, DE, USA) equipped with a film tension clamp with the same dimensions as previously defined. A stress of 0.1 MPa was applied for 10 min at 70 °C, then the stress was immediately released, and the sample was left to recover for another 30 min. This procedure was repeated every 10 °C, up to 120 or 160 °C. The viscosity η was calculated using eq 2:
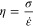
2

The deformation rate  was determined as the slope of the linear fit of the linear part of the variation of the strain as a function of time. The Angell fragility plot was then obtained plotting η as a function of *T*_g_·*T*^–1^, and the topology freezing temperature *T*_v_ can be obtained as the temperature at which the material reaches a viscosity of 10^12^ Pa·s.

### 2.10 Mechanical Recycling

The mechanical recycling was carried out by cutting into small pieces a cured sample and introducing the pieces into a round steel mold covered with Teflon to avoid the adhesion to the mold. Then, the mold was introduced in a hot-press (Schwabenthan Polystat 300 S, Berlin, Germany) preheated at 140 °C, and a pressure of 0.4 MPa was applied for 1 h. The recycled sample was manually cut into prismatic specimens for DMA analysis.

### 2.11 Chemical Recycling

Two different methodologies for chemical recycling of vitrimers were used: degradation of Cyst-BVGE/TREN cured samples through acid hydrolysis and thiol–disulfide exchange reaction. The acid hydrolysis of the imine bonds was carried out following a reported procedure.^33^ In a typical example, 0.39 g of a cured sample of Cyst-BVGE/TREN was immersed into 30 mL of a 0.2 M HCl solution in a mixture of H_2_O:THF (2:8). The sample was let to react at room temperature with magnetic stirring. After 24 h, the sample was completely solubilized. The cleavage of the disulfide bond through the thiol–disulfide exchange reaction was carried out following a reported procedure.^34^ In a typical experiment, 0.34 g of a cured sample of Cyst-BVGE/TREN was immersed into 30 mL dithiothreitol (DTT) solution in DMF at a concentration of 0.3 M. The sample was left to react at 50 °C under stirring. After 4 h of reaction, the sample was completely solubilized.

### 2.12 Composite Preparation

The composite material was prepared using the Cyst-BVGE/IPDA formulation prepared as described in Section 2.4. The carbon fiber (twill 2 × 2, 600 g·m^–2^), with 7 × 7 cm dimensions, was placed onto a Teflon sheet, and approximately 5 g of formulation was poured onto the carbon fiber. The formulation was spread out over the surface with the help of a spatula and with occasional heating with a heat gun to decrease the viscosity of the mixture. The process was repeated with two more carbon fibers with the same dimensions, and the fibers were piled up. Then, another Teflon sheet was placed over the formulation, and it was placed in a hot-press (Schwabenthan Polystat 300 S, Berlin, Germany) preheated at 80 °C and pressed at 1 MPa for 1 h. This first hour at 80 °C was used to decrease the viscosity of the mixture and to allow the formulation to permeate through all the fibers of the material. Then, the temperature was increased up to 100 °C for 2 h, 140 °C for 2 h, and finally, 160 °C for 1 h, maintaining 1 MPa of pressure, to completely cure the sample. Once completely cured, the sample was left to cool down, and the excess resin was cut off to obtain a square sample.

### 2.13 Scanning Electron Microscopy

Environmental scanning electron microscopy (FESEM) by means of an FEI Quanta 600 microscope (Thermo Fisher Scientific, Waltham, MA, USA) was used to analyze the morphology of carbon fibers before and after chemical degradation of the composites. Electrons were accelerated at 20.00 kV, and the working distance was contained between 3 and 7 mm, and ETD or T2-high-resolution secondary electron detectors were used.

### 2.14 X-Ray Photoelectron Spectroscopy (XPS)

XPS measurements were performed with the XPS machine (ProvenX-NAP), using a X-ray (AlKα – 1486.7 eV) monochromatic (μ-FOCUS 600) source. The beam spot size at sample position is 300 μm of diameter. The data were acquired with a Phoibos 150 NAP electron energy analyzer with a 1D-DLD detector, pass energy of 30 eV, entrance slit of 7 × 20 mm, and exit slit open with mesh.

### 2.15 Adhesion

Samples of steel DP1200 (ArcelorMittal, Luxemburg, Luxemburg) of 100 × 25 × 2 mm were used as adherents in single-lap shear adhesion tests, according to the EN 1465:2009 standard. The surfaces of the substrates were prepared following the EN 13887:2004 standard. Overlapping regions of 12.5 mm were degreased with acetone to remove any greasy impurities. Then, mechanical abrasion with P180 sandpaper was performed by roughening the bond area. Surfaces were cleaned with acetone again to remove any abrasion residue. Copper wires were used to ensure a bond line thickness of 0.2 mm. In addition, a constant pressure of 3 kPa was applied on the joints during curing to ensure good contact between adherents and adhesive. The final strength of the single-lap joints was evaluated by tensile lap shear tests according to the standard EN 1465:2009. The tests were performed in a universal testing machine, Lloyd EZ50 (Bognor Regis, UK), equipped with a 50 kN load cell and at 1.3 mm·min^–1^ crosshead speed. Five samples of each formulation were tested, and the average lap-shear stress was calculated.

The procedure for debonding consisted of placing the single-lap joint samples at 140 °C for 1 h and then, as the vitrimer adhesive was relaxed, disassembling by hand. In the case of self-welding, a 0.1 mm-thick layer of vitrimer adhesive was applied in both adherent plates and cured separately as previously stated. The procedure for readhesion was as follows. Both parts of the single-lap joint sample were put together (either after failure, after debonding, or for self-welding specimens). A constant pressure was applied to both halves, and the thickness of 2 mm was ensured by the presence of the copper wire. The assembly was placed at 140 °C for 1 h for readhesion.

## 3 Results and Discussion

### 3.1 Synthesis of Cystamine Bis(vanillin glycidyl ether) (Cyst-BVGE) Monomer

The first approach for obtaining the Cyst-BVGE was synthesizing the imine derivative of vanillin with cystamine (free base) and consequent glycidylation with an excess of epichlorohydrin. The imine derivative of vanillin with cystamine was already described in the literature as a curing agent for diisocyanate monomers.^35^ Although the first step of the imine formation was achieved in good yield, the second step for the glycidyl ether incorporation was unsuccessful due to the partial hydrolysis of the imine bond and the formation of species from the ring opening of the epoxide, which was observed in the ^1^H NMR spectrum (not shown). We suspect that the reaction conditions involving aqueous sodium hydroxide in a second step for the ring-closing were unsuitable in the presence of imine and disulfide groups. Therefore, the order of the reactions was inverted, and first was prepared the glycidyl derivative of vanillin, and in the second step, the imine formation with cystamine. Although a competitive reaction between the epoxides and the amine groups could also occur, the imine can be synthesized at lower temperatures than those needed for the ring opening of epoxides with amines without a catalyst.

**Scheme 2:**
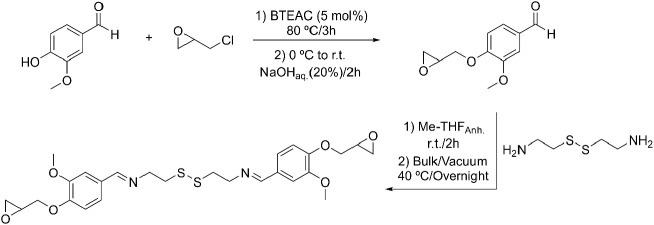
Synthesis of Cystamine Bis(vanillin glycidyl ether) (Cyst-BVGE) in a Two-Step Protocol

Then, Cyst-BVGE was prepared through a two-step procedure (Scheme 2). First, a glycidyl ether derivative of vanillin (VGE) was synthesized, followed by the condensation reaction with cystamine (free base) to obtain the imine derivative Cyst-BVGE. Both products were characterized by ^1^H and ^13^C NMR spectroscopy (Figures S1–S4) and by electrospray ionization–mass spectroscopy (ESI-MS) (Figures S5 and S6).

The VGE intermediate was obtained by reacting vanillin with a 5-fold excess of epichlorohydrin at 80 °C for 3 h in the presence of BTEAC. After this time, the mixture was cooled in an ice–water bath, and a 20% (w/w) NaOH aqueous solution was added dropwise to promote the ring closure. The product was obtained with a 78% yield as a white solid. The only purification needed was washing the precipitated solid with water and cold ethanol. The ^1^H NMR spectrum corroborated the complete incorporation of the glycidyl moiety by comparing the intensity of the signals of the glycidyl protons with those from the vanillin (Figure S1). The condensation reaction of cystamine with VGE was performed by adding a solution of VGE in anhydrous Me-THF to cystamine in stoichiometric amounts at room temperature and was let to react for 2 h. Then, the solvent was evaporated, and the oily viscous mixture was subjected to a high vacuum at 40 °C overnight under magnetic stirring to remove the water formed and displace the equilibrium. In this manner, a quantitative conversion was reached, obtaining a yellowish viscous product. Moreover, under these reaction conditions, no imine hydrolysis, disulfide cleavage, or epoxide ring opening was observed. The selection of Me-THF as renewable solvent was intended to make the process completely sustainable. Aside of the reagents used in the synthesis, all the steps involved in the preparation of the monomer should be considered green for a true biobased monomer.

### 3.2 Study of the Curing Process of Cyst-BVGE with Different Amines

**Figure 1:**
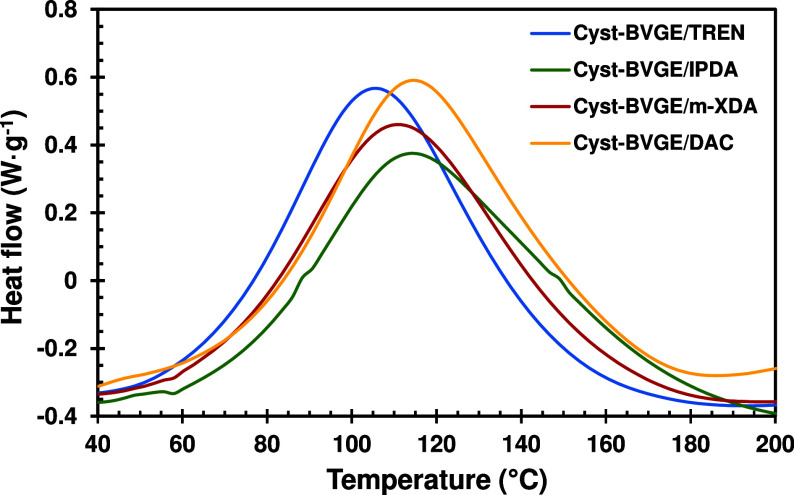
Superposed DSC thermograms of curing temperatures for the formulations of Cyst-BVGE with TREN (blue), IPDA (green), *m*-XDA (red), and DAC (yellow).

The curing reaction of Cyst-BVGE with the amine curing agents was studied by DSC. Figure 1 represents the DSC thermograms of each formulation, and the data obtained is collected in Table 2. All formulations showed a single broad curing peak, with no significant difference between the amines. The enthalpy released in kJ/eq in all formulations is quite similar, indicating that the degree of curing achieved is comparable. Moreover, the enthalpy released is near 100 kJ·eq^–1^, which is the value reported for epoxy-amine reactions.^36^

**Table 2:** Calorimetric Data of the Curing Process and Temperature of 2% of Weight Loss in an N_2_ Atmosphere of all the Formulations Studied

formulation	*T*_peak_^a^ (°C)	Δ*H*^b^ (J·g^–1^)	Δ*H*^c^ (kJ·ee^–1^)	*T*_g_^d^ (°C)	*T*_2%_^e^ (°C)	*T*_max_^f^ (°C)	char yield^g^ (%)
Cyst-BVGE/TREN	105	297	87	99	245	323	32.8
Cyst-BVGE/IPDA	114	297	93	102	240	326	27.5
Cyst-BVGE/*m*-XDA	112	284	86	97	236	326	38.9
Cyst-BVGE/DAC	117	282	84	98	222	328	27.7

a Temperature of the maximum of the exotherm of the epoxy-amine reaction.

b Enthalpy released during curing by gram.

c Enthalpy released by an epoxy equivalent.

d Glass transition temperature of the final cured material.

e Temperature of 2% of weight loss in N_2_.

f Temperatures at the maximum rate of degradation.

g Char residue at 600 °C.

The glass transition temperature (*T*_g_) was determined by DSC, showing similar values in all the formulations. In all cases, the *T*_g_s were around 100 °C (Table 2, Figure S7). Although the thermosets obtained when using the conventional DGEBA epoxy monomer cured with the same amine curing agents proposed in this study present higher *T*_g_, the values obtained here are not that far away. For instance, DGEBA/IPDA, *T*_g_ = 124 °C, and DGEBA/*m*-XDA, *T*_g_ = 105 °C.^37,38^ This led us to consider that the biobased Cyst-BVGE monomer can compete with the commercial nonrenewable monomers.

Moreover, by comparing the *T*_g_s in all the formulations, it is evidenced that a higher degree of cross-linking of the trifunctional but flexible amine (TREN) can be compensated in difunctional amines with a more rigid backbone (IPDA, *m*-XDA, and DAC). The high *T*_g_ obtained using this cystamine-based monomer contrast with lower *T*_g_s obtained (53 °C) when using cystamine as hardener in the curing of epoxy-based monomers.^39^

### 3.3 Thermal Characterization

**Figure 2:**
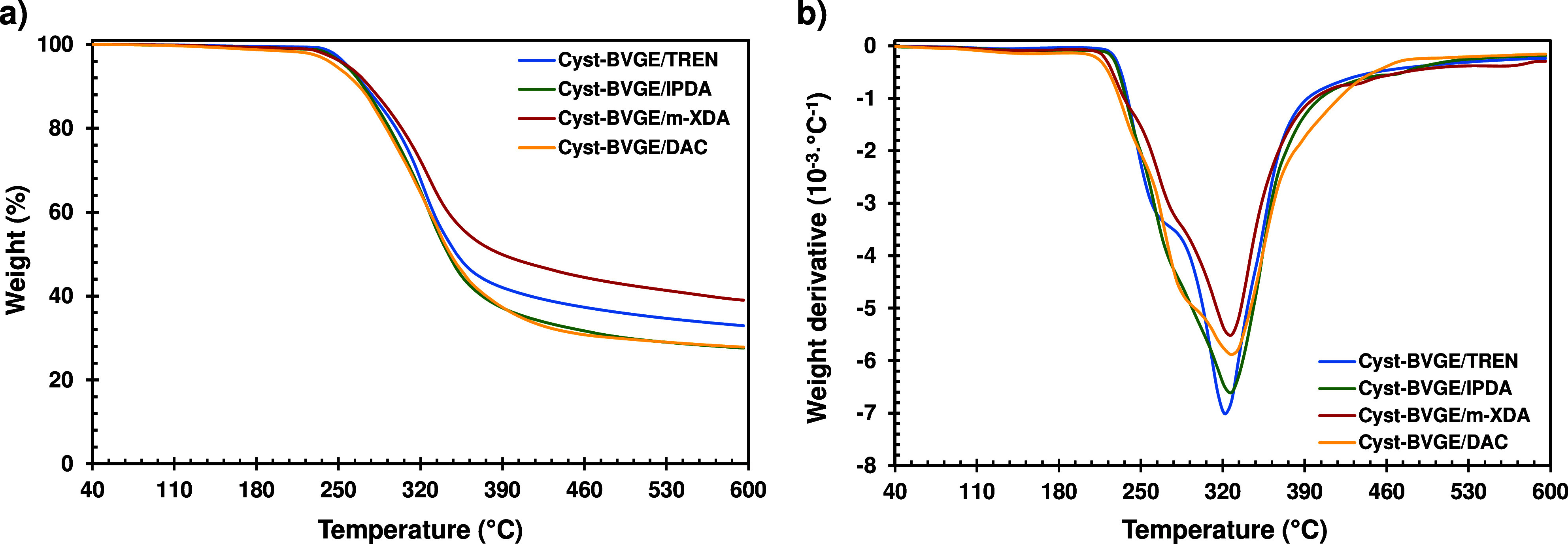
(a) TGA curves and (b) TGA 1st derivative curves of Cyst-BVGE cured samples with different amines.

The thermal stability of the cured samples was studied by thermogravimetric analysis (TGA). Figure 2a shows the TGA curves of each formulation, and Figure 2b shows the first derivative of the TGA curves for each formulation. All the thermal degradation data are summarized in Table 2. A shoulder can be observed in all the derivative curves, which could correspond to the degradation of the more labile bonds such as disulfides.^34^ The temperature of the maximum rate of degradation and the temperature of 2% weight loss (*T*_2%_) are very similar in all the samples, ranging from 323 to 328 °C and 222 to 245 °C, respectively. The most noteworthy difference regarding the thermal stability between the different formulations is the char yield obtained at 600 °C. The formulation with higher char yield is when using *m*-XDA as a curing agent, followed by TREN, and finally, DAC and IPDA are the formulations with lower char yield. These differences can be explained by the typical higher char yield obtained when aromatic groups are present,^40^ and when the material presents a higher degree of cross-linking.^41^ Unlike *m*-XDA, DAC and IPDA lack aromatic groups and have lower cross-linking density than TREN, giving the lower char yield. Regarding the *T*_2%_, these are higher than any standard polymer operating temperatures and, as will be discussed later, these materials showed complete stress relaxation at relatively low temperatures (up to 160 °C). Figure S8 shows the weight loss curve at 160 °C isotherm of a Cyst-BVGE/TREN formulation. After 3 h test, there is no significant weight loss (∼0.7%), so it can be stated that these materials are thermally stable up to this temperature.

### 3.4 Thermomechanical Characterization

**Figure 3:**
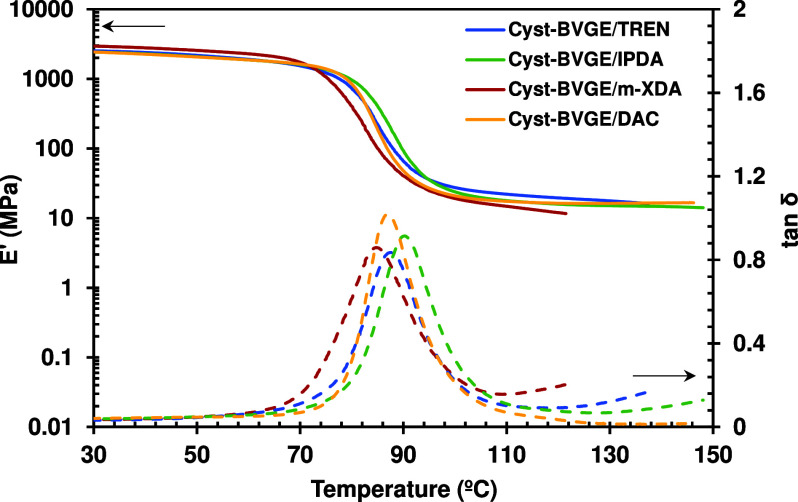
*E*′ modulus and tan δ as a function of temperature for the materials prepared.

Thermomechanical properties were determined by DMA. Figure 3 shows the storage modulus (*E*′) and tan δ as a function of temperature for the materials prepared. The main thermomechanical data obtained from those experiments are collected in Table 3. All formulations showed relatively high *T*_g_s, between 85 and 90 °C, depending on the amine curing agent. Despite the aromatic ring present in the structure of *m*-XDA and potential noncovalent interactions like π–π interactions, the Cyst-BVGE/*m*-XDA combination exhibited the lowest *T*_g_. This might be attributed to the mobility introduced by methylene units between amines and the phenylene group, which exert greater influence on molecular mobility compared to π–π aromatic–aromatic interactions, particularly with increasing temperature. As will be discussed later, this factor could significantly influence the modulus at low temperatures.

**Table 3:** Thermomechanical Data of the Thermosetting Polymers Prepared*E*_g_^′^

formulation	*T*_tan δ_^a^ (°C)	FWHM^b^ (°C)	*E*_g_^′^^c^ (MPa)	*E*_r_^′^^d^ (MPa)
Cyst-BVGE/TREN	88	14	2570	16
Cyst-BVGE/IPDA	90	13	2467	15
Cyst-BVGE/m-XDA	85	17	3015	12
Cyst-BVGE/DAC	87	12	2510	16

a Temperature of the maximum of the tan δ peak.

b Full width at half maximum.

c Storage modulus measured at *T*_g_ – 50 °C.

d Storage modulus measured at *T*_g_ + 50 °C.

On the other hand, IPDA and DAC have one and two amine groups directly attached to the cycloaliphatic moiety, respectively. Thus, Cyst-BVGE/DAC and Cyst-BVGE/IPDA showed slightly higher *T*_g_ due to the rigidity of the cycloaliphatic nature of the curing agents, which led to tighter and less mobile structures. The formulation Cyst-BVGE/TREN showed values in between due to the compensating effect between the aliphatic and flexible structure of TREN and its higher functionality that leads to a higher cross-linking density. The moduli in the glassy state were similar for samples with IPDA, DAC, and TREN, but the rigidity of the aromatic ring of the material obtained from *m*-XDA and the possible occurrence of π–π interactions increased its value. With respect to the storage modulus in rubbery state, higher functionality could lead to higher cross-linking density and thus, higher , as in the case for Cyst-BVGE/TREN. According to the theory of rubber elasticity,  is roughly proportional to the cross-linking density.^42^ However, there are other factors, such as network mobility restrictions, since these materials are far from an ideal cross-linked rubber. Thus, formulations Cyst-BVGE/IPDA and Cyst-BVGE/DAC showed the same  as Cyst-BVGE/TREN but, in these cases, is due to the tighter networks since the primary amines of its structure are directly linked to the cyclohexane, and the network mobility in the relaxed state is much more restricted. The methylene moieties of *m*-XDA (despite the benzene ring) give much more mobility to the network in the relaxed state and thus *T*_g_ and  are lower. However, as mentioned before, Cyst-BVGE/*m*-XDA showed significantly higher . The role of cross-linking density on the properties below the *T*_g_ is not clear and depends on a combination of factors such as cohesive forces and the presence of local mobility.^43^ The presence of rigid benzene rings of *m*-XDA may be the cause of this result. In any case, all formulations showed low values of fwhm (Table 2), which is indicative of a homogeneous and mobile structure^44^ and, ultimately, is beneficial for vitrimer materials to show short relaxation times.^45^

### 3.5 Vitrimeric Behavior

**Figure 4:**
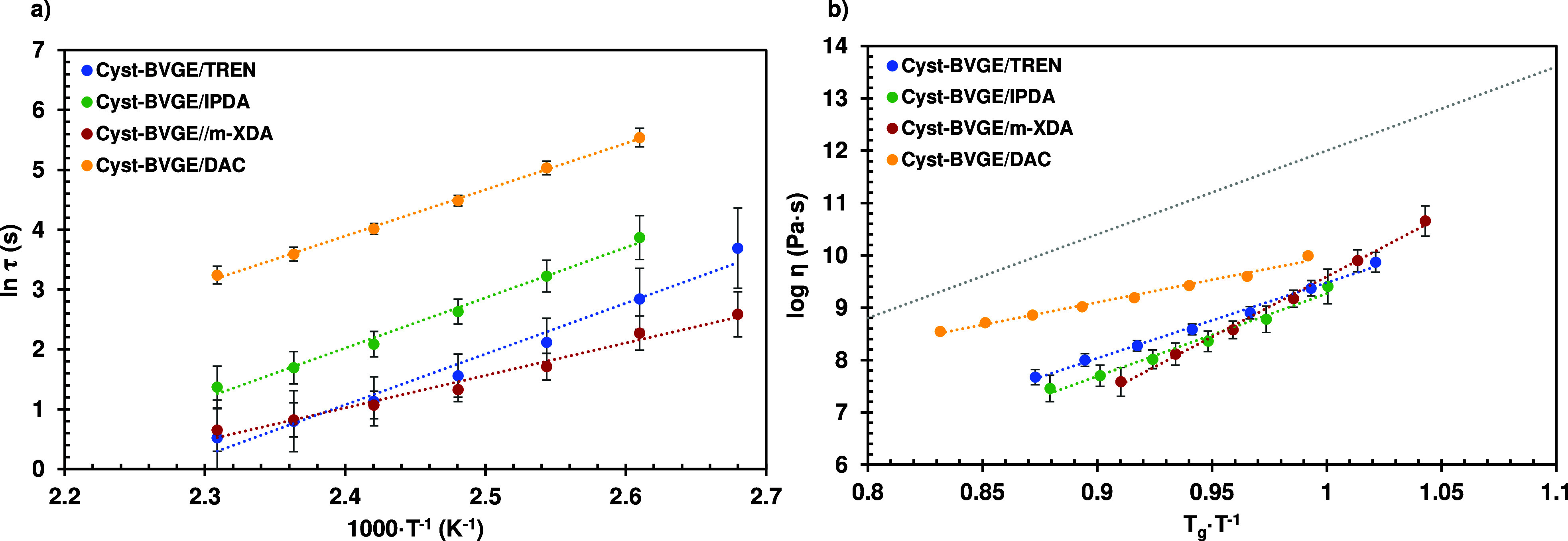
(a) Fitting of stress relaxation results to the Arrhenius equation for each formulation studied. (b) Angell fragility plot of the logarithm of the viscosity as a function of *T*_g_·*T*^–1^. For comparative purposes, an ideal strong liquid is included as a reference (gray line).

The vitrimeric behavior of the final materials was asserted by means of stress relaxation and creep tests, performed with DMA. Figure 4a shows the fitting of the stress relaxation results to the Arrhenius equation of each formulation, and Figure 4b shows the Angell fragility plot of the logarithm of viscosity as a function of *T*_g_/*T* (because *T*_g_ > *T*_v_ in these formulations). It is expected for the materials to show fast stress relaxation at high temperatures since imine and disulfide metathesis exchange reactions can occur simultaneously.^46^ In Table 4 are presented the characteristic relaxation times (τ) at 160 °C. The τ values are extremely low: formulations Cyst-BVGE/TREN and Cyst-BVGE/*m*-XDA showed τ of less than 2 s. As far as the authors of this work know, seldom have been reported such combination of thermo-mechanical and vitrimeric properties. Even in those works in which two exchange mechanisms were combined,^16,19,21^ the excellent combination of thermomechanical and vitrimeric properties presented in this work was not obtained.

**Table 4:** Characteristic Relaxation Times at 160 °C, Topology Freezing Temperature (*T*_v_) Calculated from the Angell Fragility Plot, Activation Energy, and Arrhenius Adjusting Parameters of Each Formulation

sample	τ (s)	*T*_v_ (°C)	*E*_a_ (kJ·mol^–1^)	ln *A* (s)	*R*^2^
Cyst-BVGE/TREN	1.7	34 ± 5	71 ± 13	19.3 ± 3.8	0.9758
Cyst-BVGE/IPDA	3.9	35 ± 9	70 ± 9	18.1 ± 2.6	0.9918
Cyst-BVGE/*m*-XDA	1.9	51 ± 4	45 ± 7	12.0 ± 2.1	0.9814
Cyst-BVGE/DAC	25.6	11 ± 4	64 ± 4	14.7 ± 1.1	0.9982

Figure S9 shows the stress–relaxation curves of each formulation at different temperatures. All formulations showed complete relaxation between less than 1 and 10 min at low temperatures. It has been reported in several research articles that disulfide and imine metathesis exchange reactions lead to fast stress relaxation times. Ruiz de Luzuriaga’s group reported several studies regarding epoxy-based vitrimers with disulfide metathesis exchange reaction.^47−49^ High glass transition temperatures, beyond 100 °C, and short relaxation times, below 1 min in most cases, were systematically obtained, as long as other functionalities, such as flame retardancy or shape memory. Roig et al.^8,10,50^ published excellent results with vanillin-based epoxy vitrimers with disulfide and imine exchange reactions, in which the authors obtained *T*_g_ around 100 °C and relaxation times of ∼1 min but at higher temperatures (around 180 °C). Memon et al.^7,51^ synthesized imine-containing amine and phenol curing agents from vanillin, which were used to prepare epoxy vitrimers. The *T*_g_ of the final materials and the stress relaxation times depended on the epoxy monomers used. Although this is an interesting strategy for preparing epoxy-based vitrimers, we think that the approach introduced in the present work allows for more versatility since the exchange mechanisms are included in the epoxy monomer.

From Figure 4, it can be observed that the vitrimeric behavior entirely depends on the curing agent selected. It has been reported that transimination follows an associative mechanism,^52^ meanwhile the mechanism of disulfide metathesis is ambiguous or follows a combination of dissociative and associative mechanisms.^53^ Here, the type of mechanism resulting from combining both exchange reactions depends on the curing agent. According to DMA experiments (Figure 3), it seems that there was no plateau in  at high temperatures for formulation Cyst-BVGE/m-XDA, which is indicative of loss of structural integrity (that is, dissociative mechanism). On the contrary, in the case of formulation Cyst-BVGE/DAC, it is an associative mechanism since a plateau in  is observed at *T* > *T*_g_. More ambiguous are the behaviors of formulations Cyst-BVGE/TREN and Cyst-BVGE/IPDA, in which a slight decrease is observed as temperature increases. Notwithstanding, it has been proved that the viscosity–temperature relationship of CANs with dissociative mechanisms can also fit Arrhenius equation,^54^ as can be observed in Figure 4a. Therefore, these materials can be considered as vitrimer-like materials. This can be observed in the Angell fragility plot (Figure 4b), in which all formulations showed viscosity values much lower than an ideal strong liquid. The strain–time creep plots of all formulations from 80 to 160 °C are shown in Figure S10. It can be observed that there is no significant increase in the strain over time at *T* < *T*_g_. However, from *T* = *T*_g_, the strain rate rapidly increases with temperature. This behavior could be explained by the structures of the amine curing agents: the higher functionality of TREN, the aromatic structure of *m*-XDA, and small cycloaliphatic structure of IPDA and DAC all have the same effect, which hinder the molecular motion of the materials and prevent them to show creep at service temperature. Once the temperature exceeds the *T*_g_ threshold, the double relaxation mechanism imparts the materials with a low viscosity.

This serves to widen our previous studies of effective mechanism to control the creep behavior of vitrimers.^55^ However, in this study, only four different amine curing agents were used, and a deeper study of the effect of the curing agent on vitrimeric behavior must be carried out. For instance, Ruiz de Luzuriaga et al.^47,56^ demonstrated that the regioisomerism of the primary amine with aromatic disulfide moieties may lead to different relaxation times. This could explain the differences between Cyst-BVGE/IPDA and Cyst-BVGE/DAC, which can be assumed to be similar curing agents, but still they showed significant different behavior.

The activation energies (*E*_a_) obtained correlated to those reported in the literature for materials with imine (80 kJ·mol^–1^) and disulfide (55 kJ·mol^–1^) groups.^57^ Being closer to the disulfide *E*_a_ as it has higher activation energy and, therefore, it would be the rate-determining step.

### 3.6 Mechanical Recycling

**Figure 5:**
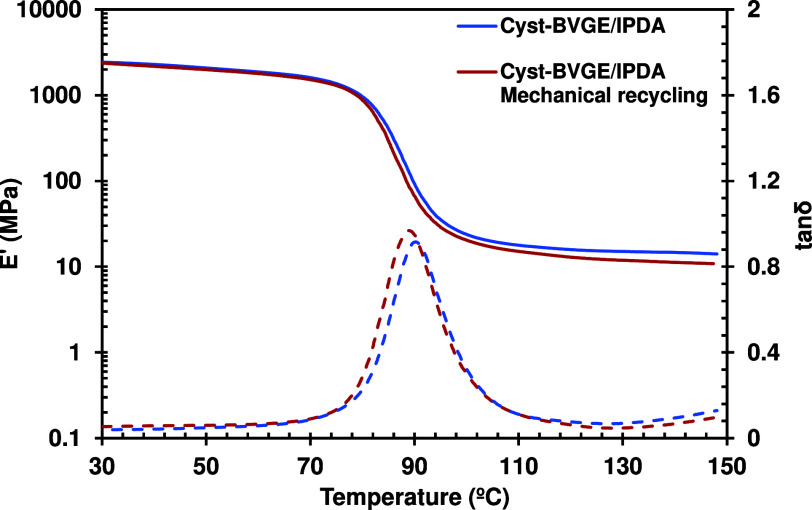
Storage modulus *E*′ and tan δ as a function of temperature of pristine Cyst-BVGE/IPDA formulation and after mechanical recycling.

The fast stress relaxation times shown by these formulations encouraged us to perform the mechanical recycling of the Cyst-BVGE/IPDA sample under mild conditions. The temperature used for the recycling was relatively low (140 °C) with a relatively low pressure of 0.4 MPa. The sample was cut into small pieces, but it was not necessary to ground them into small particles, as under this temperature, the vitrimer can easily flow. The photographs of the recycled sample (Figure S12) showed the joints of the pieces, evidenced by darker regions in the sample. However, the sample looked homogeneous. Compared with pristine formulation, there were no significant changes in thermomechanical properties, according to DMA characterization (Figure 5). The only remarkable difference is a slight decrease in , probably due to the cutting process that may destroy some of the permanent bonds. It should be noted that the mechanical recycling process was not optimized in temperature, time, and/or pressure.

### 3.7 Chemical Degradation

The chemical degradation of cured samples was performed through two different mechanisms: the acid hydrolysis of the imine bonds and the disulfide cleavage through a thiol–disulfide exchange.

**Scheme 3:**
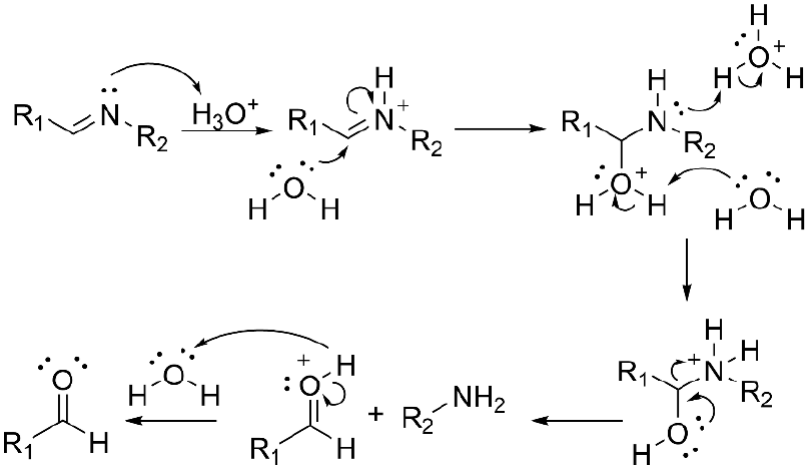
Mechanism of Imine Acid Hydrolysis

The acid hydrolysis of the imine moieties is a well-known mechanism, where the acid protonates the lone pair electrons on the nitrogen of the imine group, and the aldehyde (or ketone) and amine are obtained after the nucleophilic attack of water (Scheme 3).^58^

**Scheme 4:**
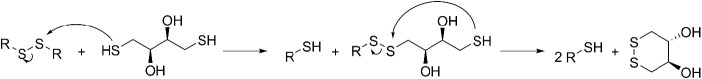
Thiol–Disulfide Exchange Reaction Using Dithiothreitol (DTT) as Reagent

On the other hand, the thiol–disulfide exchange reaction is also a well-known reaction. The nucleophilic attack of a thiol to the sulfur on a disulfide moiety gives rise to an equilibrium of a mixture of the disulfide of the thiol added and the thiol released from the original disulfide. For this reason, when using monothiols in this reaction (e.g., 2-mercaptoethanol), a great excess of thiol is used. However, when using dithiothreitol (DTT), the formation of a highly stable six-membered cyclic disulfide drives the reaction toward the formation of products (Scheme 4).^59^ Another advantage of using DTT instead of other thiols is that it is a solid at room temperature and considerably less odorous than the typical thiols.^60^

#### 3.7.1 Degradation by Acid Hydrolysis

**Scheme 5:**
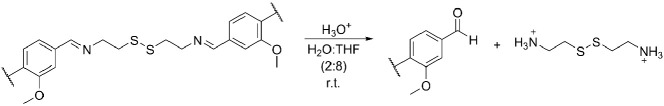
Acid Hydrolysis of Imine Groups on Cyst-BVGE/TREN Cured Samples

**Figure 6:**
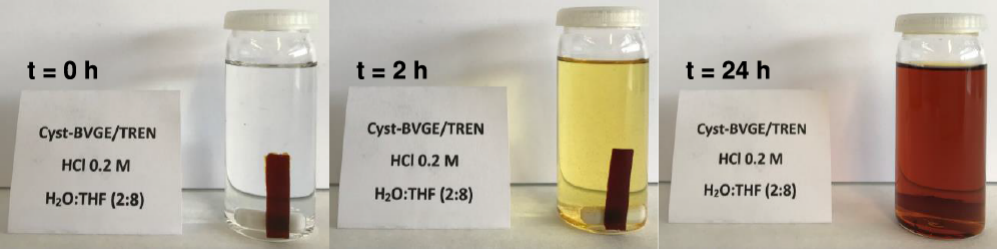
Photographs of cured Cyst-BVGE/TREN sample immersed into a 0.2 M HCl solution in an H_2_O:THF (2:8) mixture at *t* = 0, 2, and 24 h.

**Figure 7:**
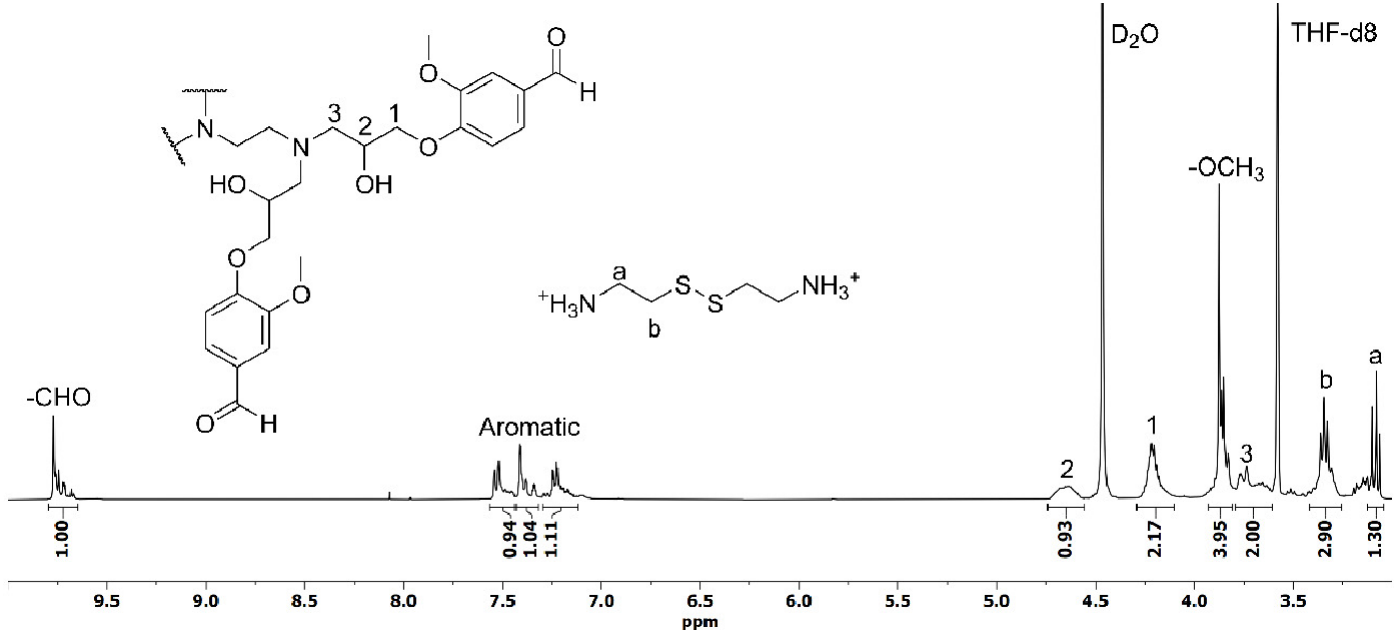
^1^H NMR spectrum, section from 3.0 to 10.0 ppm, of acid degraded Cyst-BVGE/TREN sample using DCl (0.2 M) solution in D_2_O:THF-*d*_6_ (2:8).

The acid hydrolysis of the imine bonds of cured samples (Cyst-BVGE/TREN) was carried out by submerging the sample into a 0.2 M solution of HCl in an H_2_O:THF (2:8) mixture at room temperature, following previously reported procedures (Scheme 5).^33^ The specimen was left to degrade with magnetic stirring, and photographs of the progress of the degradation were taken over time (Figure 6). After 24 h, the material was solubilized entirely into the mild acidic medium, obtaining a dark-colored solution. To determine the structure of the products formed, the reaction was repeated in deuterated solvents, as well as deuterium chloride solution as the acid. The reaction was performed directly in an NMR tube and analyzed once the sample was completely solubilized. In the ^1^H NMR spectrum (Figure 7), it can be observed the appearance of aldehyde signals whose integration correlates with the aromatic protons in 1 to 3 ratio and the signals corresponding to the open glycidyl group (signals 1 to 3 in Figure 7). This indicates that the degradation only occurs in the imine moieties. Moreover, it can be observed the appearance of the signals corresponding to the cystamine salt (signals a and b in Figure 7).

**Figure 8:**
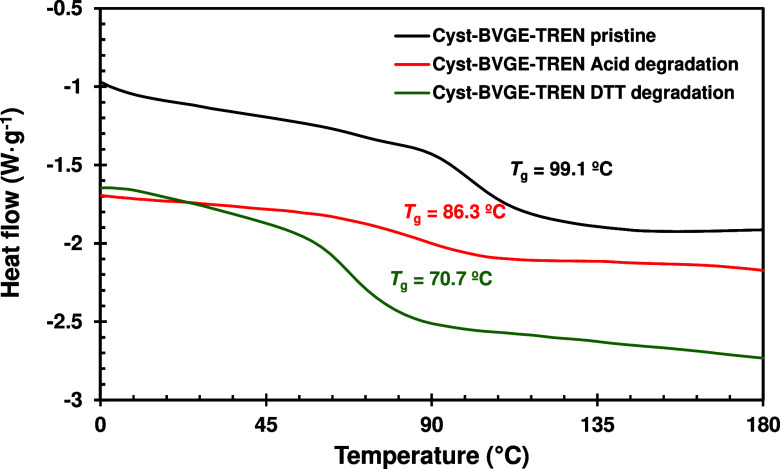
Superposed DSC thermograms of Cyst-BVGE-TREN formulations before degradation (black) and after recycling the acid degraded sample (red) and the DTT degraded sample (green).

The degraded sample was neutralized with a 2 M sodium hydroxide solution and evaporated to dryness to test its feasibility of recovering the material. The evaporated sample was poured into a mold and introduced into a vacuum oven at 50 °C for 3 h, 130 °C for 2 h, and 160 °C for 1 h to obtain the chemically recycled material. The appearance of bubbles in the sample was observed since the formation of the new imine groups caused the release of water. However, the obtained recycled material possesses a *T*_g_ similar to the original, as the sample was analyzed by DSC and compared with the *T*_g_ of the original sample (red curve in Figure 8).

#### 3.7.2 Cleavage by Thiol–Disulfide Exchange

**Scheme 6:**
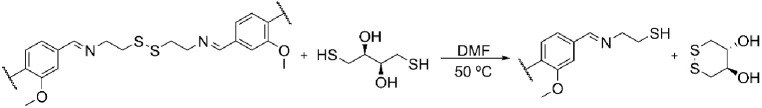
Disulfide Cleavage on Cyst-BVGE/TREN Cured Samples Using DTT

**Figure 9:**
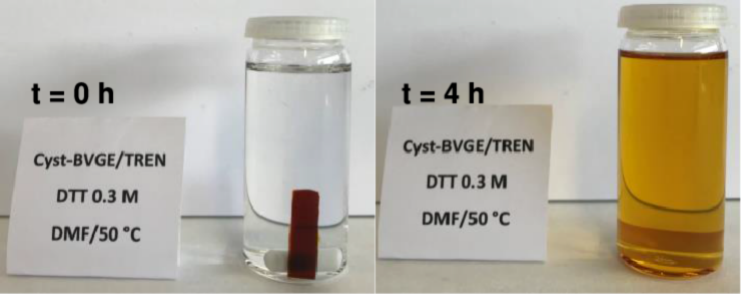
Photographs of cured Cyst-BVGE/TREN sample immersed onto a 0.3 M DTT solution in DMF at 50 °C at *t* = 0 and 4 h.

Two main methods typically perform the disulfide cleavage. One is by using reducing agents, such as tributylphosphine,^61^ and another is by using the thiol–disulfide exchange reaction.^62^ Tributyl phosphine has some drawbacks because of its tendency to be air oxidized, volatility, and high toxicity. Moreover, the reduction of disulfides using tributyl phosphine is known to be an irreversible reaction due to the high stability of the tributyl phosphine oxide formed.^60^ Instead, by using dithiols, such as dithiothreitol (DTT), for the thiol–disulfide exchange, the reaction can be reversed to reform the disulfide bond. The use of DTT allows a faster exchange than by using monothiols due to the stability of the cyclic disulfide formed. The reaction was carried out following a reported procedure,^34^ where a cured sample was submerged into a 0.3 M solution of DTT in DMF at 50 °C (Scheme 6). The reaction was conducted in these conditions under stirring, and photographs were taken during the progress of the reaction (Figure 9). It can be observed that after only 4 h, the sample was completely solubilized. The degradation of the disulfide bond can also be conducted at room temperature. However, the time required to completely solubilize the sample is extended from 3 days up to 2 weeks, depending on the thickness of the sample. In the test performed, the thickness of the sample was between 1.0 and 1.5 mm.

**Figure 10:**
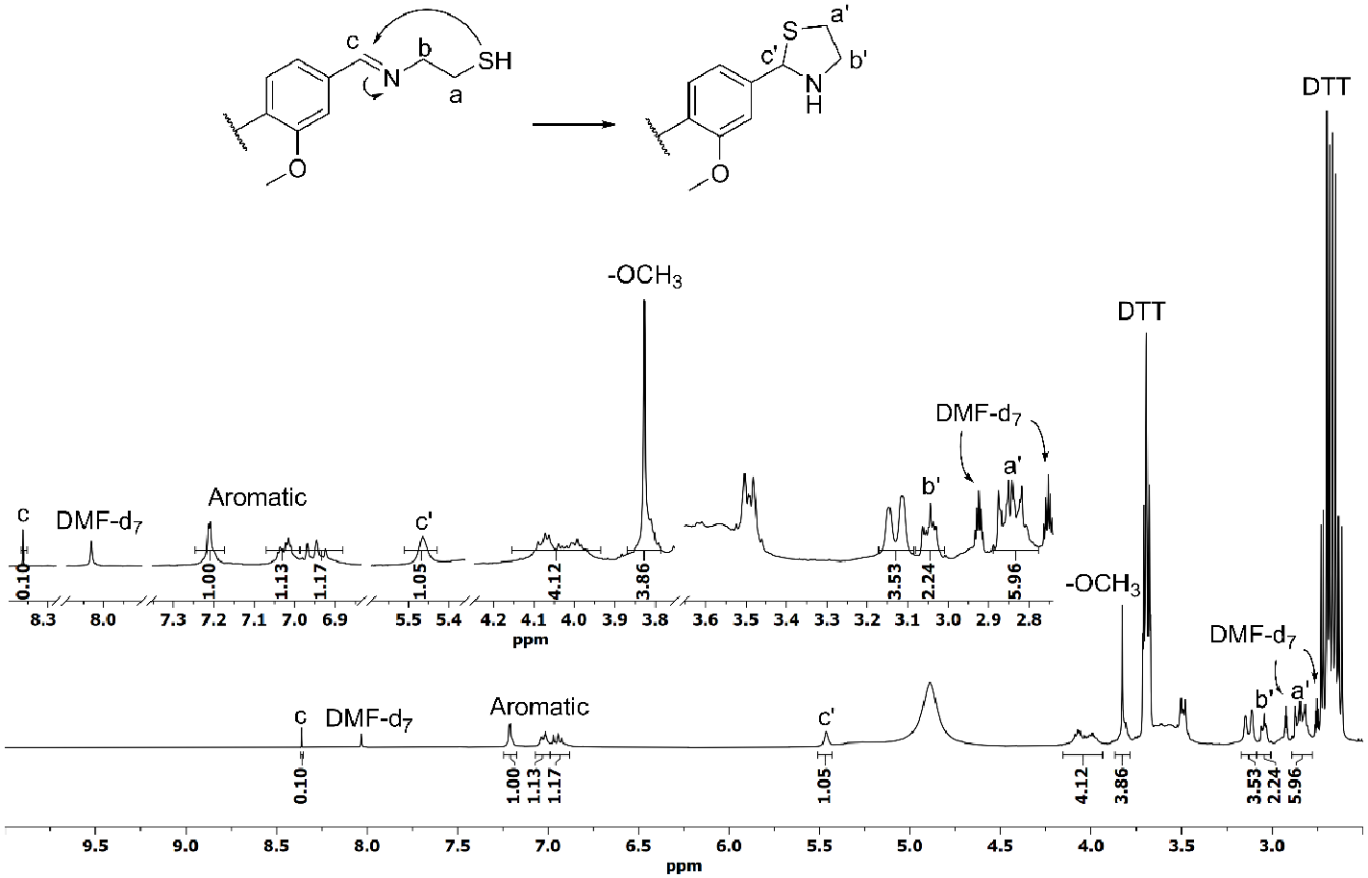
^1^H NMR spectrum, section from 2.5 to 10.0 ppm, of reduced Cyst-BVGE/TREN sample using DTT (0.3 M) solution in DMF-*d*_7_.

As in the acidic degradation, the treatment with the dithiol was repeated directly in an NMR tube using deuterated DMF as the solvent to determine the products formed during the degradation. The ^1^H NMR spectrum showed the signal corresponding to the imine proton (signal c in Figure 10); however, its intensity does not match with those of the aromatic signals. This fact could indicate a partial hydrolysis of the imine moiety during the disulfide cleavage, but no signal of aldehyde was observed. Thus, another side reaction may have been occurred. The appearance of a new signal at 5.46 ppm (signal c′ in Figure 10) indicates the formation of a thiazolidine derivative from the nucleophilic attack of the thiol, formed after the disulfide reduction, to the electrophilic carbon on the imine. The chemical shift of this signal correlates to those in the literature for similar compounds.^63^ Moreover, the intensity of the signal correlates with those of the aromatic protons. All this evidence indicates that the major product in the degradation is the thiazolidine derivative, which could lead to difficulties when reforming the recycled material.

Nevertheless, the material was tested for its reforming after chemical degradation. For this purpose, the DMF solution was concentrated under vacuum and precipitated in diethyl ether to try to eliminate the excess of DTT. After the elimination of ether, an oily product was obtained, which was subjected to the same curing cycle as the original sample. Afterward, the material presented bubbles possibly from remaining solvent, therefore only one sample for DSC analysis could be obtained. It can be observed, in the superposed DSC curves, that in the case of the disulfide degraded sample, the material obtained after recycling has lower *T*_g_ than in the case of degradation with HCl (green curve in Figure 8).

These results demonstrate that Cyst-BVGE materials can be reformed after chemical degradation to obtain a new material with similar properties. Certainly, the favorable conjunction of good thermomechanical characteristics, as detailed in Section 3.4, coupled with chemical degradability via exchange reactions, establishes a promising avenue for the incorporation of Cyst-BVGE material within recyclable composite matrices.

### 3.8 Reshaping and Recovery of Carbon Fibers in Composite Materials

**Figure 11:**
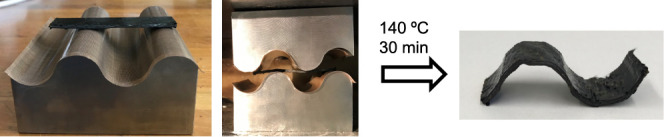
Reshaping of carbon-fiber composites through hot-pressing.

The suitability of the Cyst-BVGE/IPDA formulation as the matrix in composite materials was tested using carbon fiber. The composite was prepared as a preimpregnated (prepreg) material (Figure S12). The traditional prepregs consist of the impregnated fiber with the corresponding resin only partially cured. In this manner, the material can be reshaped in a mold and then to be completely cured with the new shape by the application of heat. The drawback of prepregs is that they must be stored in a cooled area (usually −20 °C) to avoid the premature curing of the resin. The use of a vitrimeric polymer matrix in the preparation of prepregs overcomes this drawback, as the resin can be completely cured within the fiber, and then, by the applying of heat, the material can be reshaped to the final shape. With this approach, it is not necessary to store the material in a cold environment. In order to demonstrate this feature, a 49 cm^2^ of multilayered carbon-fiber reinforced composite was prepared according to Section 2.12. The composite was then placed in a preheated wavy-shape mold and hot pressed at 140 °C for 30 min (Figure 11). Eventually, a reshaped composite is obtained that would not have been possible to obtain if conventional epoxy resins were used.

**Figure 12:**
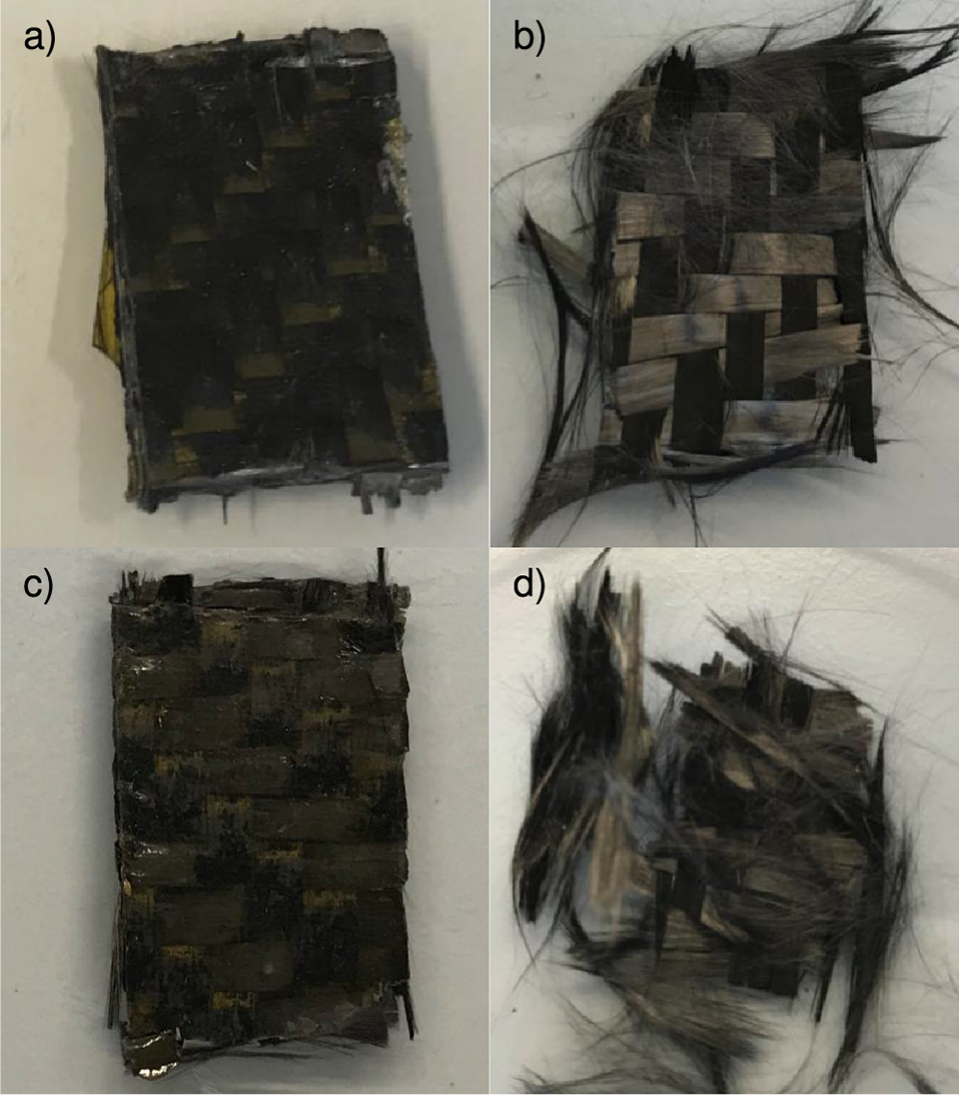
Photographs of carbon-fiber composite (a and c) before degradation, (b) after acid degradation, and (d) after thiol–disulfide exchange degradation.

Moreover, the polymer matrix of the composite could be chemically degraded for fiber recovery, which could be used to prepare a new composite. A sample of the carbon-fiber composite (Figure 12a,b) was chemically degraded by the two different methods described in Section 2.10. In a similar manner as in the case of the pure thermoset material, the composite samples were immersed into an 0.2 M HCl solution of H_2_O:THF (2:8) at room temperature under magnetic stirring for 24 h or in a 0.3 M DTT solution in DMF at 50 °C under stirring for 4 h. After this time, the fiber was removed from the solution and washed with THF or DMF. It can be observed that the resin was completely removed from the fibers (Figure 12c,d). Additionally, the carbon fibers after the composite chemical degradation were examined by FESEM, microscopy to ensure the fibers did not suffer any modification because of the degradation processes. FESEM micrographs are shown in Figures S13–S15, and it can be observed that there is not any physical change in the fibers after chemical degradation; thus, they can be employed for making a further carbon-fiber reinforced composite.

For further confirmation that the carbon fibers remain unaffected after the degradation process, the fibers were analyzed by XPS. The XPS survey spectra of the pristine carbon fiber, the carbon fiber after acid degradation, and after DTT degradation are shown in Figures S16–S18, respectively. In the case of DTT degradation, the spectrum shows no evidence of chemical modification. However, in the case of acid chemical degradation, signals corresponding to nitrogen, chlorine, and sulfur are present. We attribute these signals to remaining cystamine dihydrochloride, formed after the degradation, that were not properly eliminated during the washing of the carbon fiber. The chemical composition of the fibers is shown in Table S1, showing similar values before and after degradation. The more remarkable difference is a slightly decrease on the oxygen content (from 17.5% on the original fiber to 12.7% and 14.7% after acid and DTT degradation, respectively). This decrease in oxygen content is also described in the literature for the recovery of carbon fibers using more harsh conditions (e.g., nitric acid).^64^

### 3.9 Adhesive Performance

Another application that could benefit from vitrimer characteristics is adhesion. The adhesive performance of Cyst-BVGE was assessed through single-lap shear tests. Table 5 collects the average results of lap-shear adhesion strength of formulation Cyst-BVGE/IPDA. The values of the first adhesion are not particularly high in comparison with other epoxy-based vitrimer-like adhesives.^65,66^ Notwithstanding, the results are probably caused by poor surface preparation since the five samples failed adhesively. Very interesting results were obtained in terms of readhesion though. Almost the same values of the first adhesion were obtained with any readhesion method. In the case of readhesion after break, the results are lower because the remended joint is formed by an adhesive-steel interface (since the failure mechanism was adhesive). In the case of readhesion after debonding, the failure was mainly adhesive, although there were zones of cohesive failure. That is the reason for the slight increase of readhesion values with respect to readhesion after break. On the contrary, in the case of readhesion after self-welding, the new joint is formed by adhesive–adhesive interface, and the exchange reactions are favored. Many factors may affect the reversibility of vitrimer adhesives,^66^ but ultimately, the vitrimer properties and flowing behavior of the adhesives are the most influential for reversible adhesion. As discussed in Section 3.5, the vitrimer performance of these materials is exceptional, and this eventually leads to such values of adhesion reversibility. Another application that could benefit from vitrimer characteristics is adhesion. The adhesive performance of Cyst-BVGE was assessed through single-lap shear tests. Table 5 collects the average results of lap-shear adhesion strength of formulation Cyst-BVGE/IPDA. The values of the first adhesion are not particularly high in comparison with other epoxy-based vitrimer-like adhesives.^65,66^ Notwithstanding, the results are probably caused by poor surface preparation since the five samples failed adhesively. Very interesting results were obtained in terms of readhesion though. Almost the same values of the first adhesion were obtained with any readhesion method. In the case of readhesion after break, the results are lower because the remended joint is formed by an adhesive-steel interface (since the failure mechanism was adhesive). In the case of readhesion after debonding, the failure was mainly adhesive, although there were zones of cohesive failure. That is the reason for the slight increase of readhesion values with respect to readhesion after break. On the contrary, in the case of readhesion after self-welding, the new joint is formed by adhesive–adhesive interface, and the exchange reactions are favored. Many factors may affect the reversibility of vitrimer adhesives,^66^ but ultimately, the vitrimer properties and flowing behavior of the adhesives are the most influential for reversible adhesion. As discussed in Section 3.5, the vitrimer performance of these materials is exceptional, and this eventually leads to such values of adhesion reversibility.

**Table 5:** Average Lap-Shear Strength of the First Adhesion and Rejoined Samples of Formulation Cyst-BVGE/IPDA

	lap-shear strength (MPa)			
	first adhesion	readhesion after break	readhesion after self-welding	readhesion after debonding
Cyst-BVGE/IPDA	7.3 ± 0.6	6.3 ± 1.6 (86.2%)	7.2 ± 3.1 (97.5%)	6.7 ± 1.2 (90.9%)

The same methodology of chemical recycling was used with single-lap shear adhesion plates with the remains of adhesives (Figure S19). The adhesive stuck to the plates was degraded by dipping the plate in the 0.3 M DTT solution in DMF (50 °C, 4 h). Vitrimeric adhesives represent a move forward in terms of sustainability in adhesive joints. The combination of joint dismantling and chemical degradation of the vitrimers allows recycling the metal substrates, which are often high-added value materials, so that they can be used in another joint or can be easily cleaned from adhesive remains and used for another purpose.

## 4 Conclusions

In this study, a new epoxydic monomer having two imine and one disulfide groups has been synthesized from renewable vanillin and cystamine in a two-step protocol, with an easy procedure, obtaining excellent yields. The synthesized monomer was cured using different amines, obtaining vitrimeric materials with high *T*_g_s (around 100 °C) that showed extremely fast relaxation times at relatively low temperatures, which allows reshaping composite materials or joint dismantling in case of adhesive applications.

The materials showed chemical degradation under mild conditions in an acid medium and by a thiol–disulfide exchange reaction. The chemical degradation products were characterized by ^1^H NMR spectroscopy to demonstrate the selectiveness of both methods, and the materials were reformed from the degradation products, giving rise to new materials with similar properties. The chemical degradability allows not only the recyclability of the polymeric material but also the recovery of the adherent when used as an adhesive or the fiber when used for composite materials. The presence of the two dynamic exchangeable groups within the same monomer allows great versatility in the curing agent used, as the vitrimeric and degradation behavior would be minimally affected while the thermal and mechanical properties could be tuned by varying the curing agent.

Hence, a material with outstanding properties could be prepared with relatively cheap compounds derived from renewable resources together with a sustainable procedure by using green solvents. This material has great potential both as an adhesive and as the matrix in composite materials, as it has great advantages compared to the common adhesives and polymeric matrices, such as the dismantling and readhesion or the reshaping of composite materials and storing at room temperature prepregs. The vitrimeric behavior allows not only the reusability but also the recyclability of the polymer, the adherent, and/or the fiber by simple methods.

## Supporting Information Available

Figure S1, ^1^H NMR spectrum of VGE in CDCl_3_; Figure S2, ^13^C NMR spectrum of VGE in CDCl_3_; Figure S3, ^1^H NMR spectrum of Cyst-BVGE in CDCl_3_; Figure S4, ^13^C NMR spectrum of Cyst-BVGE in CDCl_3_; Figure S5, ESI-MS spectrum of VGE [M + H^+^]; Figure S6, ESI-MS spectrum of Cyst-BVGE [M + H^+^]; Figure S7, superposed DSC thermograms of cured samples of all formulations; Figure S8, TGA isotherm at 160 °C of a Cyst-BVGE/TREN cured sample for 3 h; Figure S9, stress relaxation curves at different temperatures of formulations Cyst-BVGE/TREN, Cyst-BVGE/IPDA, Cyst-BVGE/*m*-XDA, and (d) Cyst-BVGE/DAC; Figure S10, Strain–time plots of formulations Cyst-BVGE/TREN, Cyst-BVGE/IPDA, Cyst-BVGE/m-XDA, and Cyst-BVGE/DAC; Figure S11, photographs of a cured sample of Cyst-BVGE/IPDA virgin grinded and after mechanical recycling; Figure S12, photographs of Cyst-BVGE/IPDA formulation over carbon fiber before curing and after piling 3 carbon fibers together, curing under pressure, and after removing the excess resin; Figure S13, SEM images of pristine carbon fiber; Figure S14, SEM images of carbon fiber after acid degradation; Figure S15, SEM images of carbon fiber after thiol–disulfide exchange degradation; Figure S16, XPS spectrum of pristine carbon fiber; Figure S17, XPS spectrum of carbon fiber after acid degradation; Figure S18, XPS spectrum of carbon fiber after thiol–disulfide exchange degradation; Table S1, XPS element quantification of pristine CF and CF after HCl/DTT degradation; Figure S19, elimination of adhesive remains after debonding through thiol–disulfide exchange reaction (PDF)
