# Supplementary material for: A Biobased Epoxy Vitrimer with Dual Relaxation Mechanism: A Promising Material for Renewable, Reusable, and Recyclable Adhesives and Composites
Source: ACS Sustain Chem Eng. 2024 Apr 4;12(15):5965–78. doi: 10.1021/acssuschemeng.4c00205 (PMC11022369; doi:10.1021/acssuschemeng.4c00205)
Supplement: Supplementary file 1 — sc4c00205_si_001.pdf [file sc4c00205_si_001.pdf]

A bio-based epoxy vitrimer with dual relaxation mechanism: A promising material for renewable, reusable, and recyclable adhesives and composites

Pere Verdugo[1,2,], David Santiago[1,3], Silvia De la Flor[3], Àngels Serra[2]*

[1] Eurecat, Technology Center of Catalonia - Chemical Technologies Unit, c/Marcel·lí Domingo 2, 43007 Tarragona, Spain.

[2] Universitat Rovira i Virgili, Department of Analytical and Organic Chemistry, c/Marcel·lí Domingo 1, 43007 Tarragona, Spain.

[3] Universitat Rovira i Virgili, Department of Mechanical Engineering, Av. Països Catalans 26, 43007 Tarragona, Spain.

*Corresponding author: pere.verdugo@eurecat.org

Phone number: +34 977 29 70 17 ext. 4536

Keywords: vanillin, epoxy, vitrimers, disulfide, imine, composite, adhesion.

Number of pages: 13

Number of figures: 19

Number of tables: 1

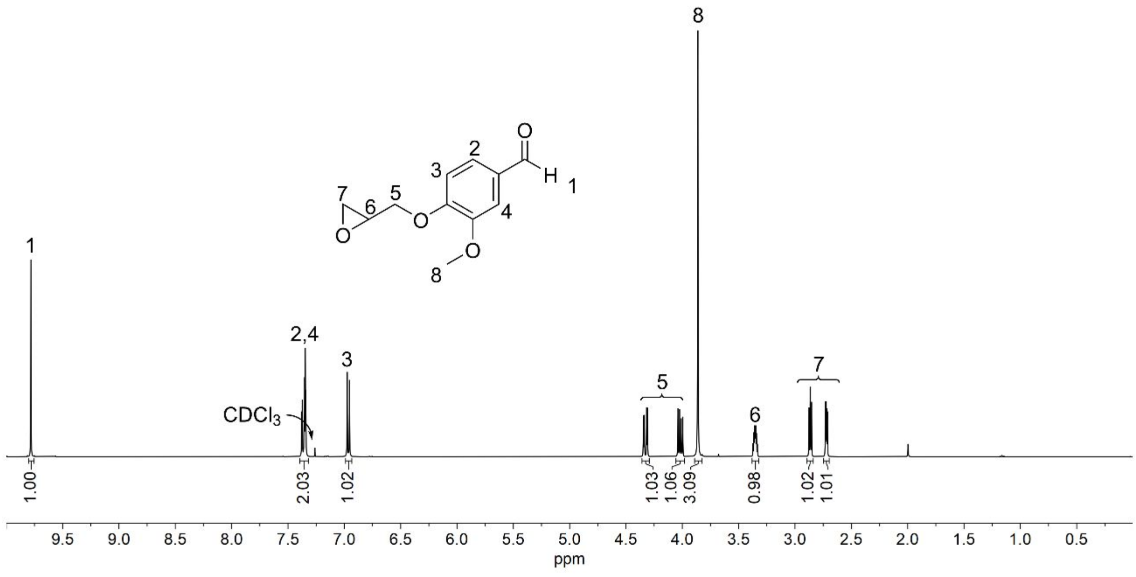

Figure S1. ^{1}H-NMR spectrum of VGE in $CDCl_3$.

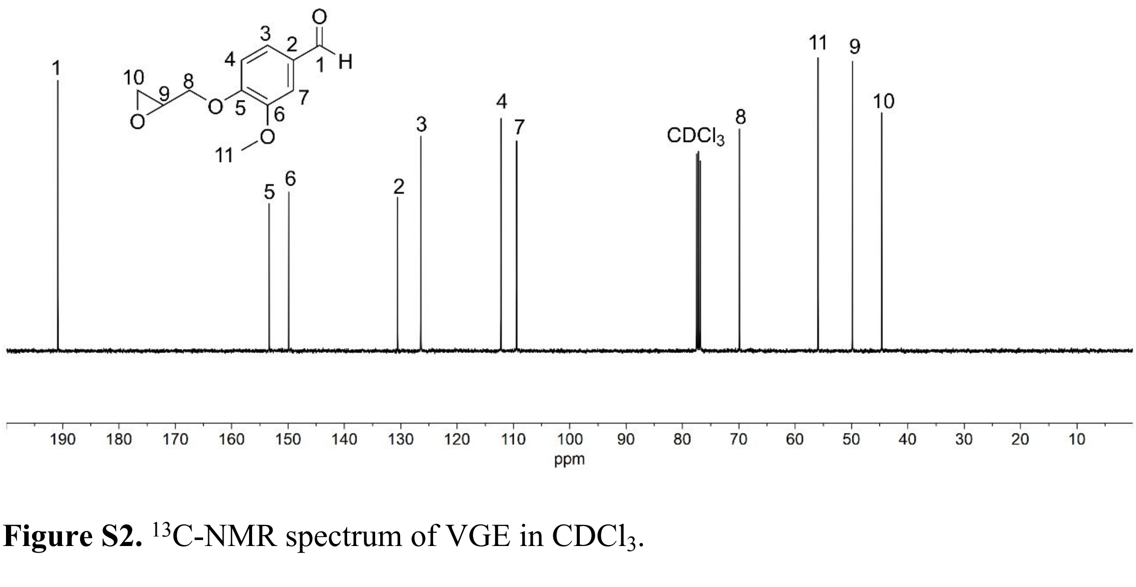

Figure S2. ^{13}C-NMR spectrum of VGE in $CDCl_3$.

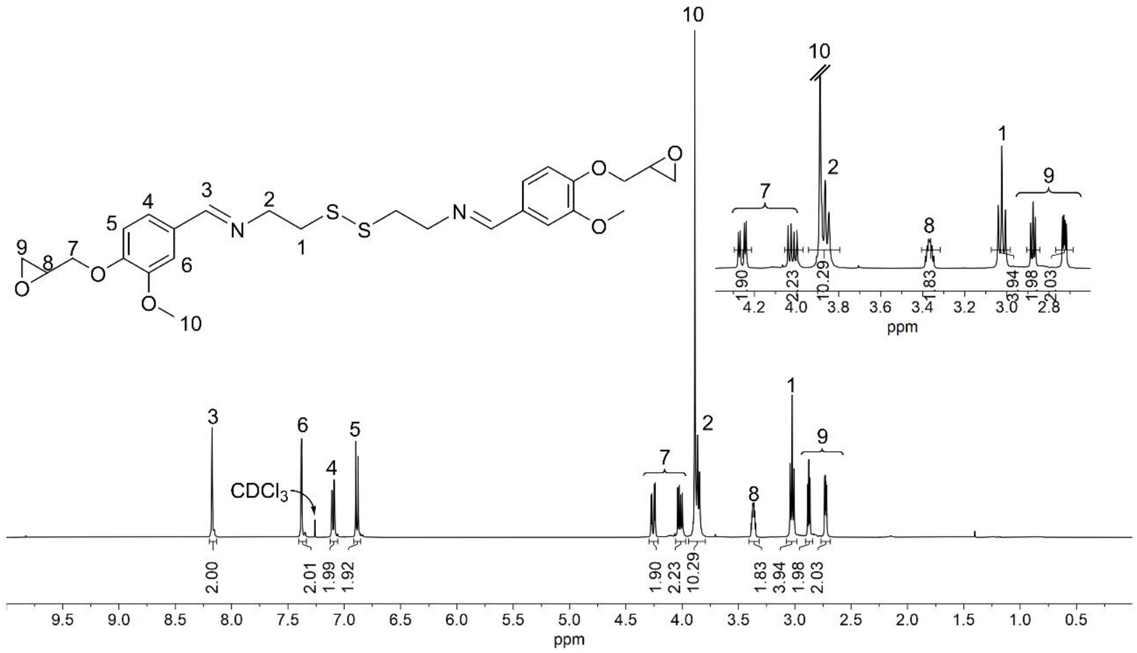

Figure S3. ^{1}H-NMR spectrum of Cyst-BVGE in $CDCl_3$.

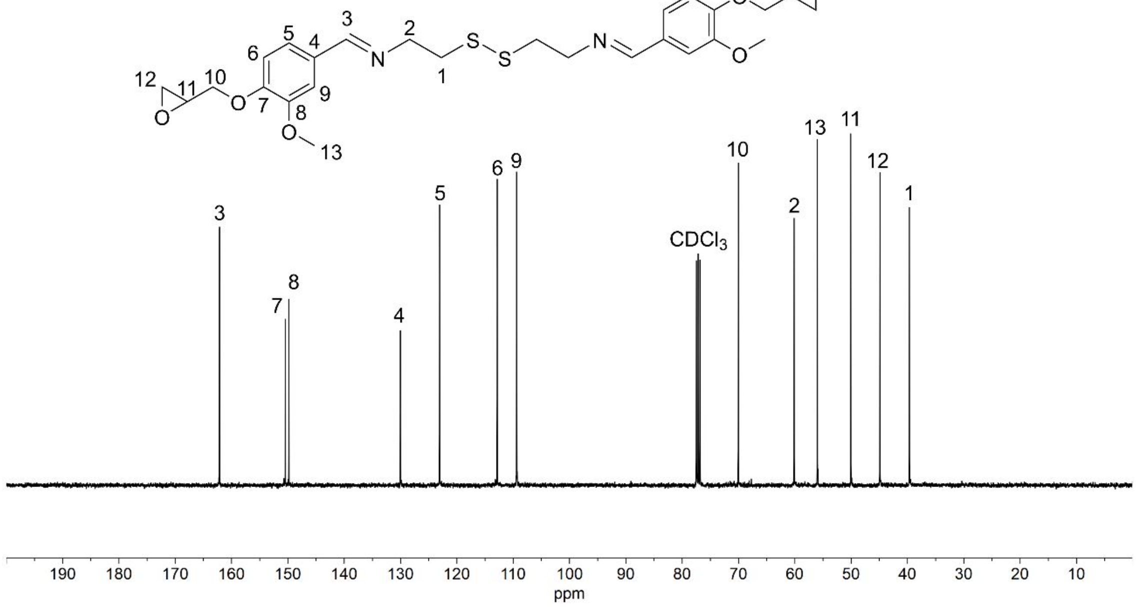

Figure S4. ^{13}C-NMR spectrum of Cyst-BVGE in $CDCl_3$.

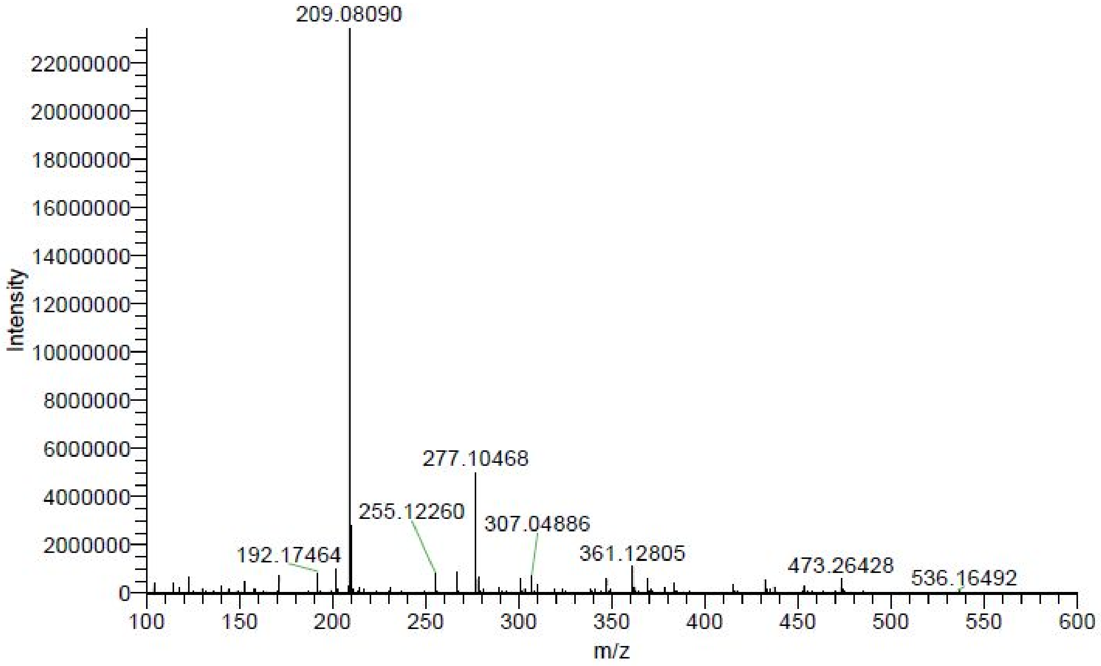

Figure S5. ESI-MS spectrum of VGE [M+H$^+$].

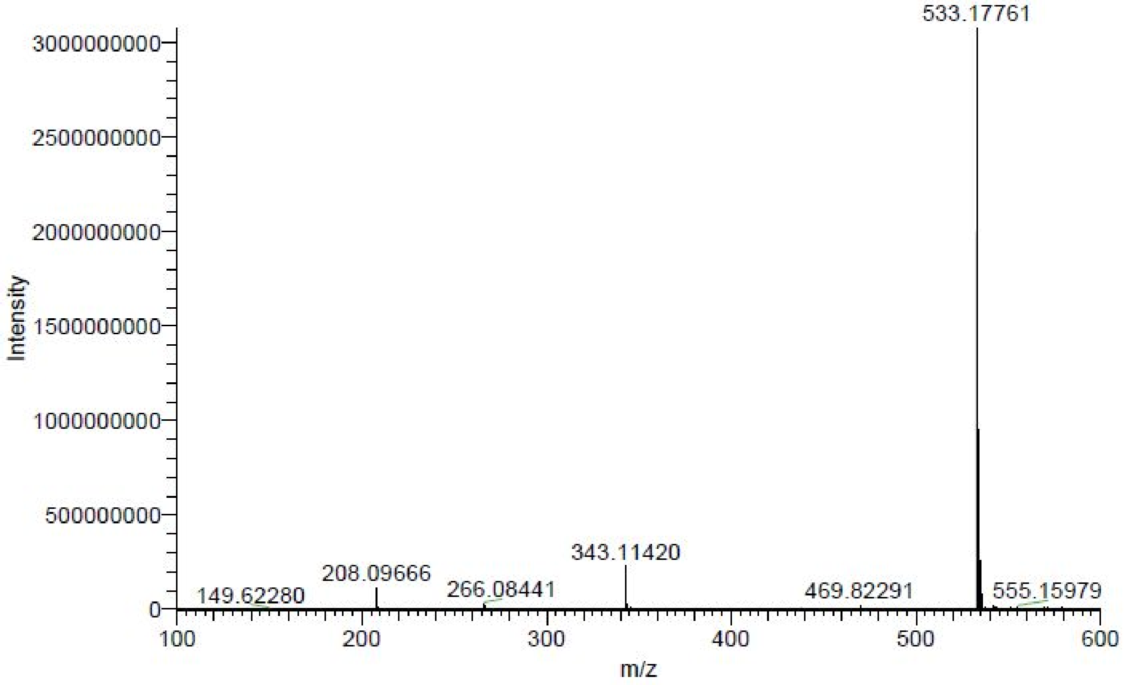

Figure S6. ESI-MS spectrum of Cyst-BVGE [M+H$^+$].

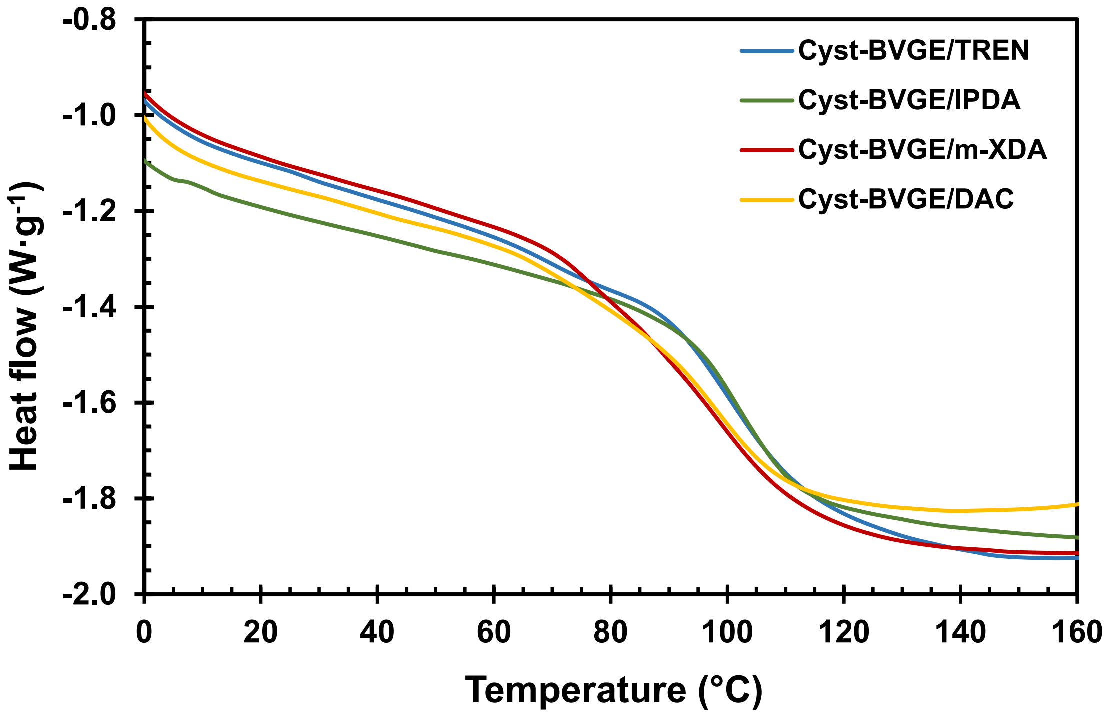

Figure S7. Superposed DSC thermograms of cured samples of all formulations.

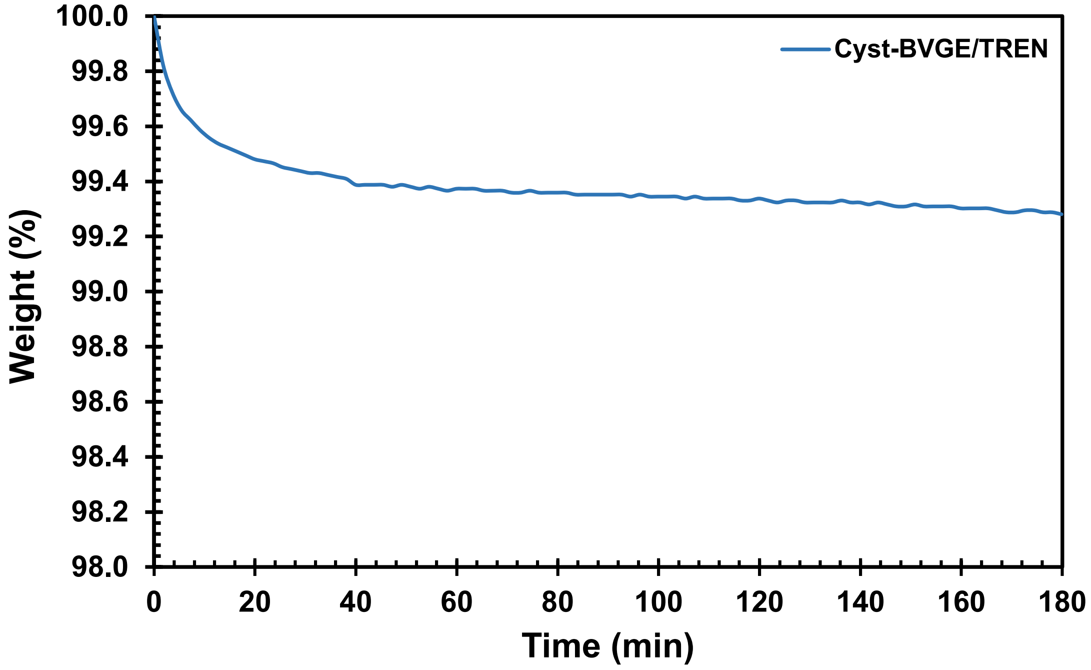

Figure S8. TGA isotherm at 160 ºC of a Cyst-BVGE/TREN cured sample for 3 hours.

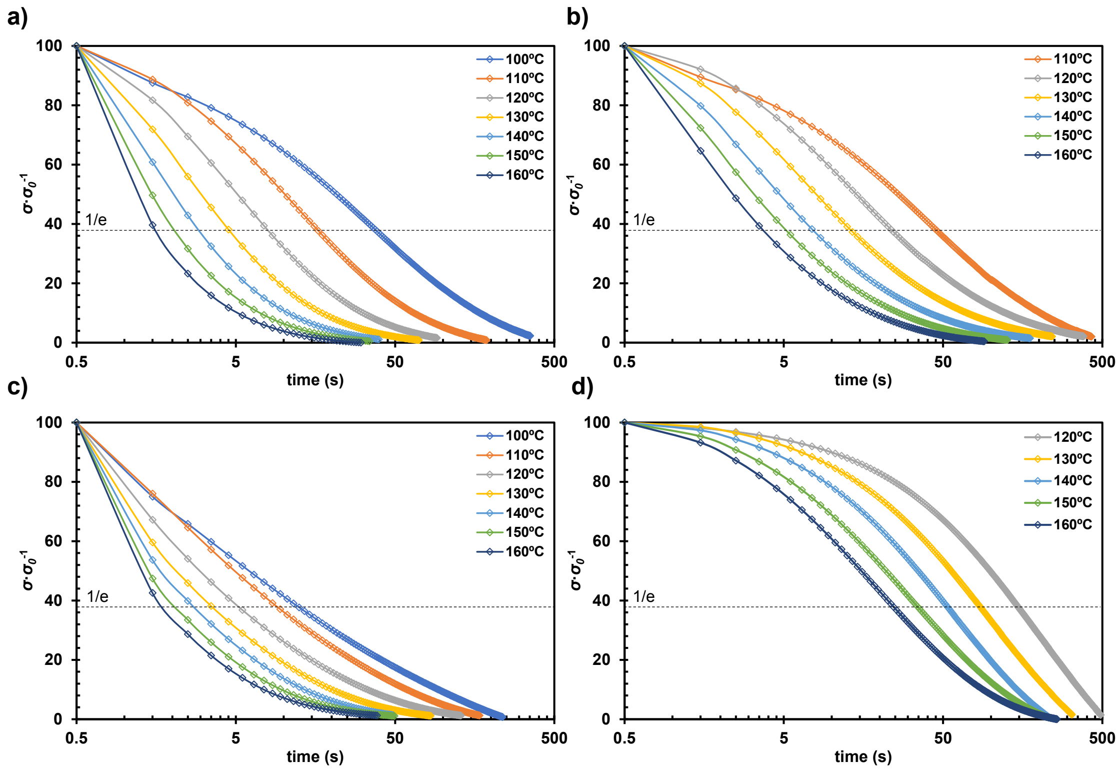

Figure S9. Stress relaxation curves at different temperatures of formulations (a) Cyst-BVGE/TREN, (b) Cyst-BVGE/IPDA, (c) Cyst-BVGE/m-XDA, and (d) Cyst-BVGE/DAC.

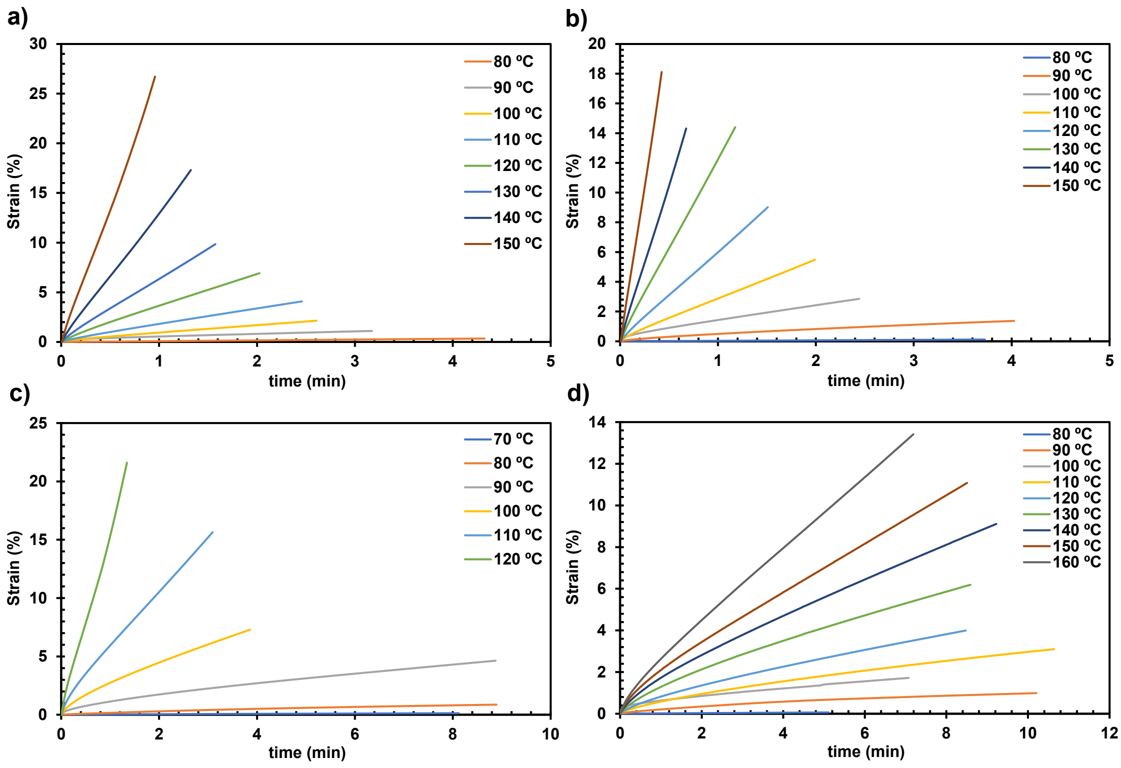

Figure S10. Strain-time plots of formulations Cyst-BVGE/TREN (a), Cyst-BVGE/IPDA (b), Cyst-BVGE/m-XDA (c) and Cyst-BVGE/DAC (d).

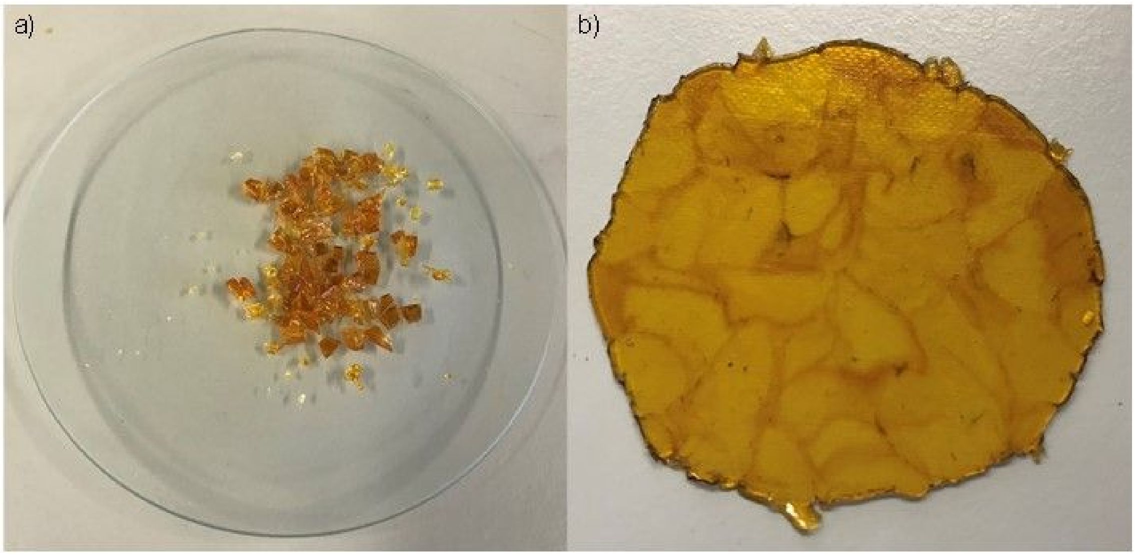

Figure S11. Photographs of a cured sample of Cyst-BVGE/IPDA (a) virgin grinded and (b) after mechanical recycling.

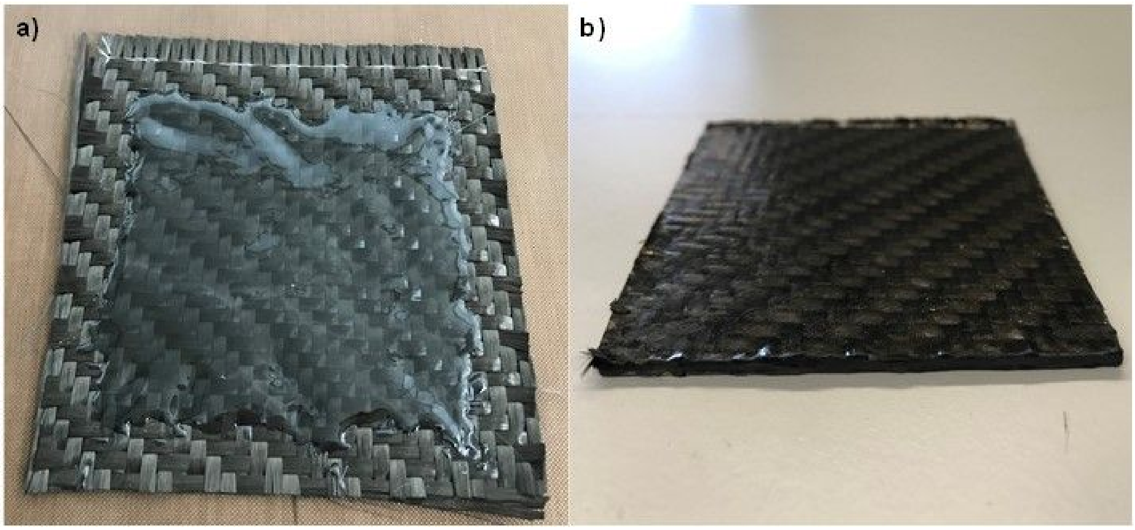

Figure S12. Photographs of (a) Cyst-BVGE/IPDA formulation over carbon fiber before curing, and (b) after piling 3 carbon fibers together, curing under pressure, and after removing the excess resin.

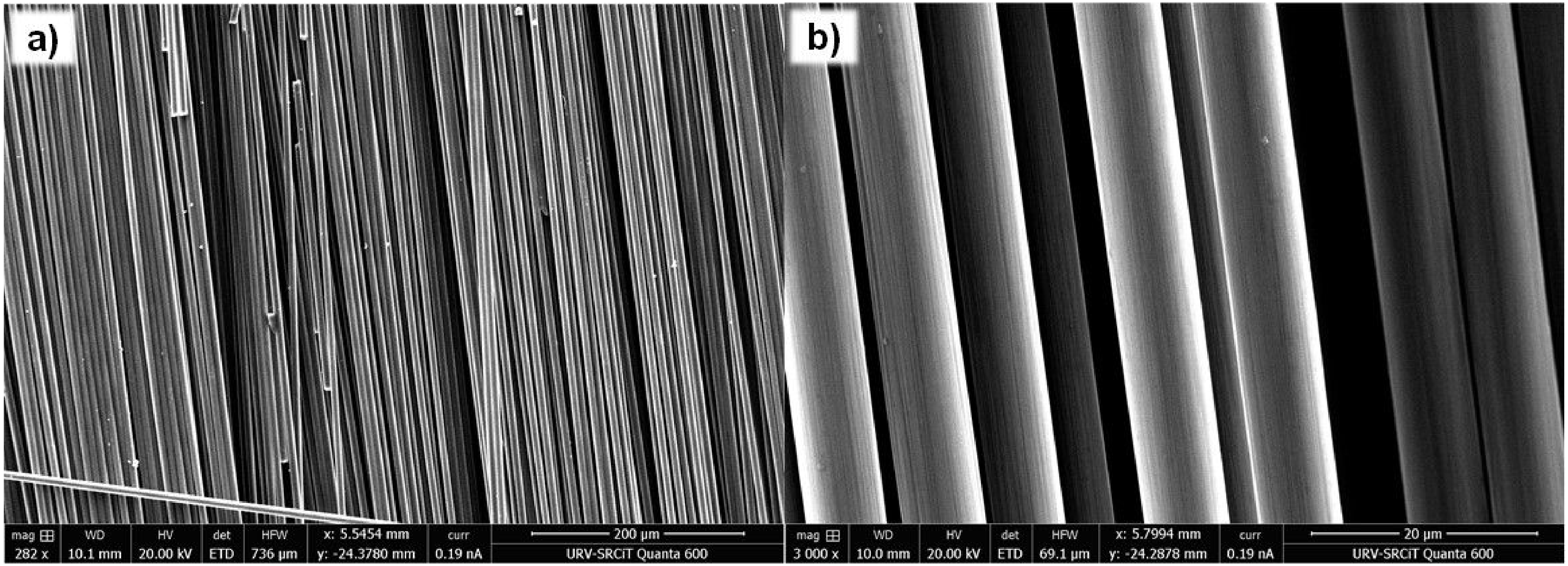

Figure S13. Scanning Electron Microscopy (SEM) images of pristine carbon fiber at (a) 282x and (b) 3000x.

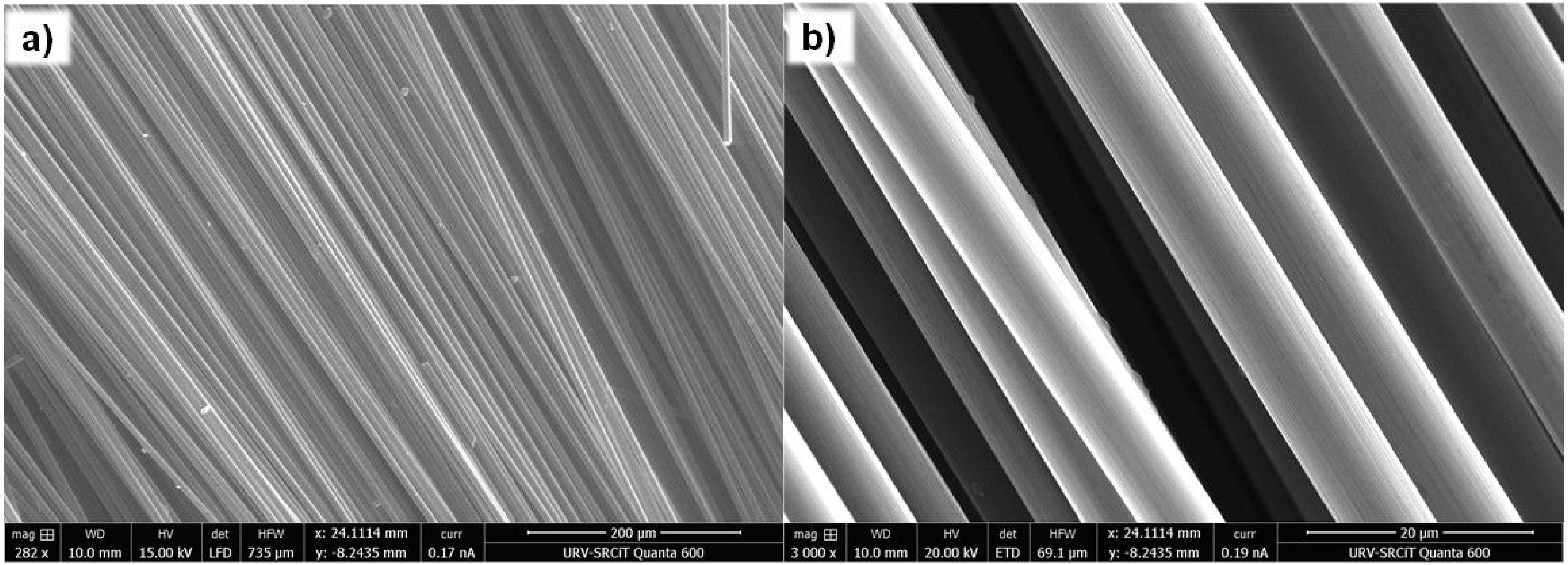

Figure S14. Scanning Electron Microscopy (SEM) images of carbon fiber after acid degradation at (a) 282x and (b) 3000x.

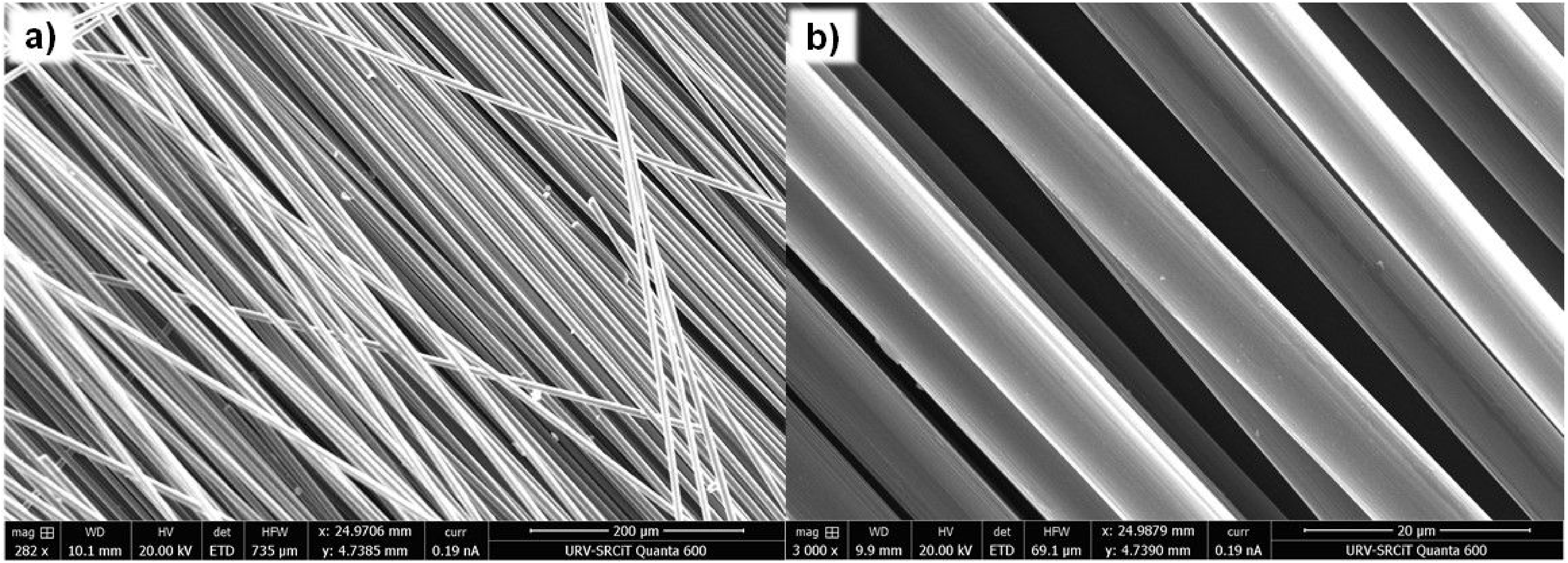

Figure S15. Scanning Electron Microscopy (SEM) images of carbon fiber after thiol-disulfide exchange degradation at (a) 282x and (b) 3000x.

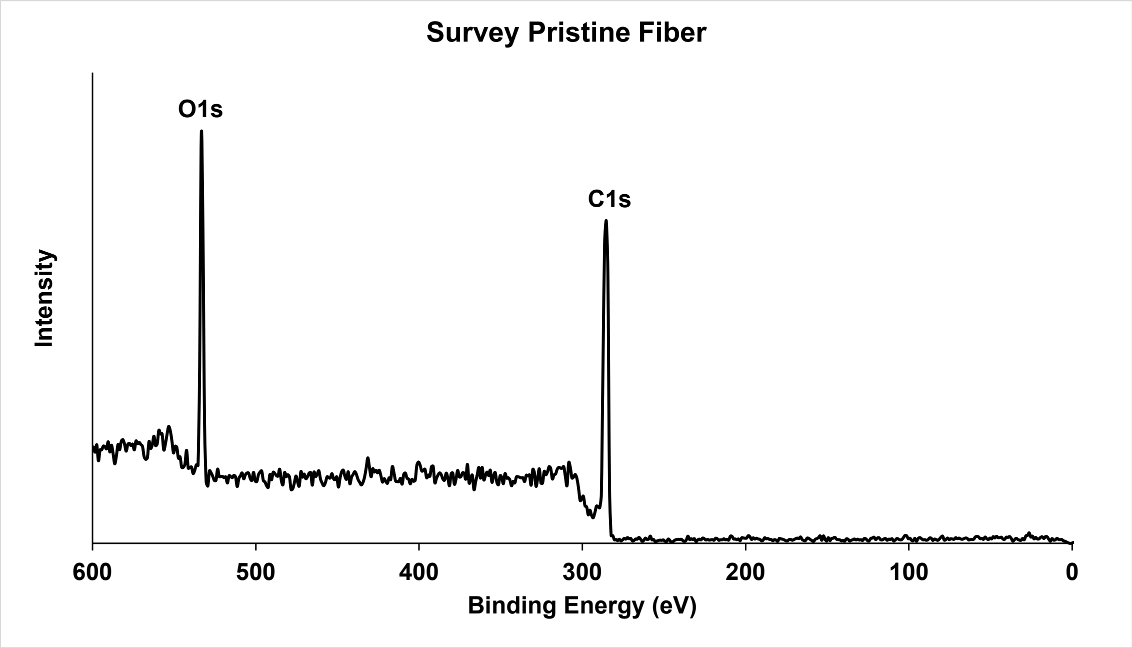

Figure S16. XPS spectrum of pristine carbon fiber.

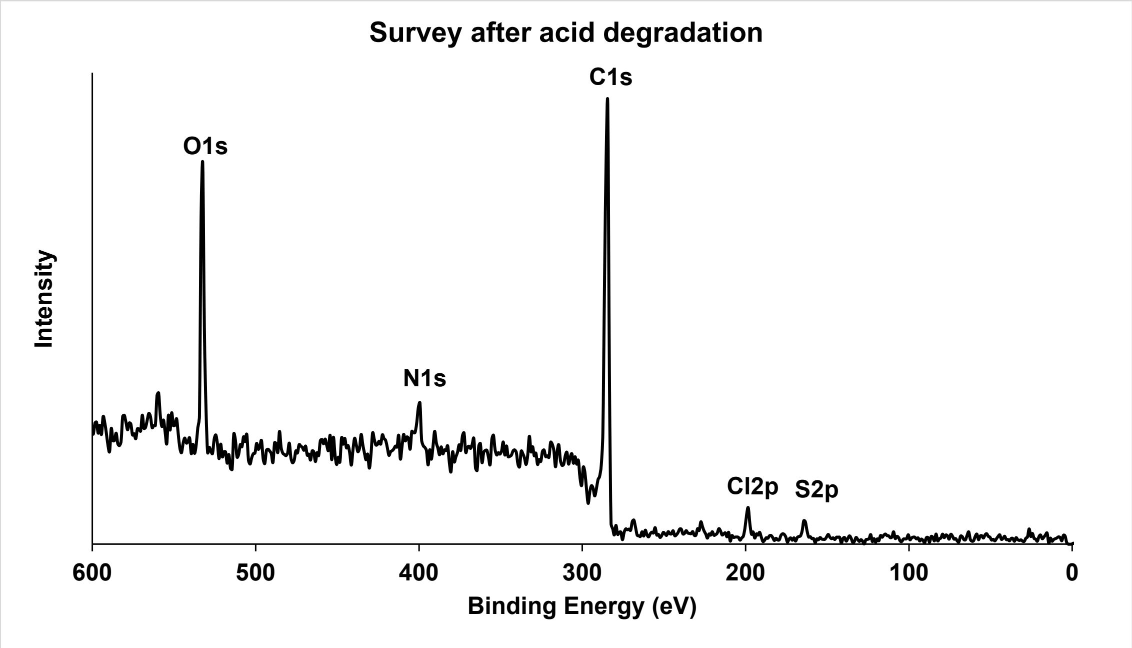

Figure S17. XPS spectrum of carbon fiber after acid degradation.

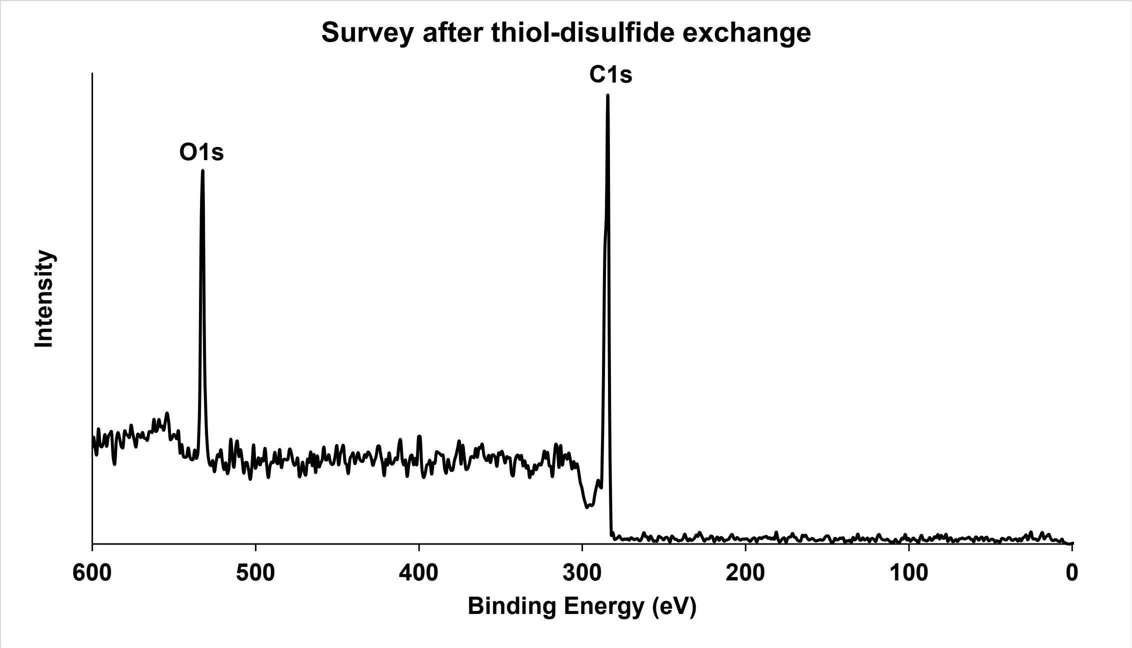

Figure S18. XPS spectrum of carbon fiber after thiol-disulfide exchange degradation.

Table S1. XPS element quantification of pristine CF and CF after HCl/DTT degradation.

Sample	C (%)	O (%)	N (%)	S (%)	Cl (%)	Others (%)
**Pristine CF**	80.0	17.5	1.7	-	0.1	0.6
**CF After HCl degradation**	80.5	12.7	3.5	1.4	1.9	-
**CF After DTT degradation**	81.2	14.7	2.6	0.8	-	0.6

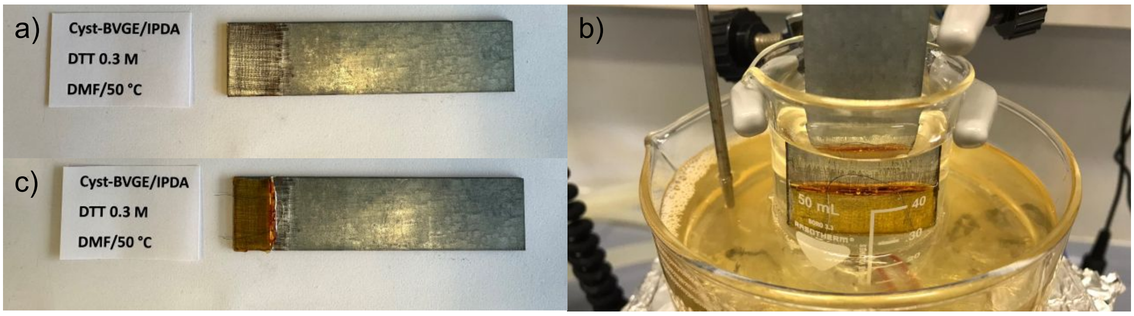

Figure S19. Elimination of adhesive remains after debonding through thiol-disulfide exchange reaction. (a) adhesive remains after joint dismantling. (b) chemical degradation

process with 0.3 M DTT solution in DMF at 50 ºC under stirring for 4 hours. (c) recycled stainless steel plates.
